# An Open Soil Health Assessment Framework Facilitating Sustainable Soil Management

**DOI:** 10.1021/acs.est.2c04516

**Published:** 2022-11-18

**Authors:** Gerard H. Ros, Sven E. Verweij, Sander J. C. Janssen, Janjo De Haan, Yuki Fujita

**Affiliations:** † Nutrient Management Institute, Nieuwe Kanaal 7C, 6709 PA Wageningen, Netherlands; ‡ Environmental System Analysis Group, Wageningen University and Research, P.O. Box 47, 6700AA Wageningen, the Netherlands; § Environmental Research, Wageningen University and Research, P.O. Box 47, 6700AA Wageningen, the Netherlands; ∥ Plant Research, Wageningen University and Research, P.O. Box 16, 6700AA Wageningen, the Netherlands

**Keywords:** holistic soil health assessment, agricultural fields, sustainable crop production, valorization, open-source framework, farming practices

## Abstract

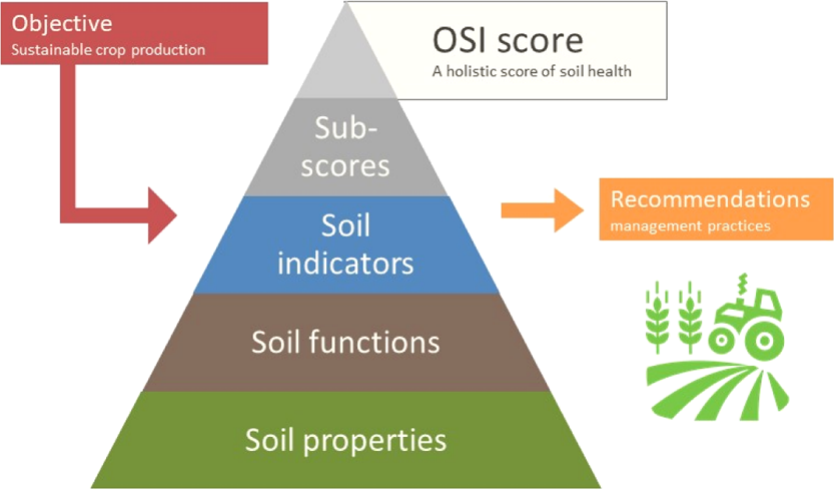

The crucial role of healthy soil in achieving sustainable food production and environment is increasingly recognized, as is the importance of proper assessment of soil quality. We introduce a new framework, open soil index (OSI), which integrally evaluates soil health of agricultural fields and provides recommendation for farming practices. The OSI is an open-source modular framework in which soil properties, functions, indicators and scores, and management advice are linked hierarchically. Soil health is evaluated with respect to sustainable crop production but can be extended to other ecosystem functions. The OSI leverages the existing knowledge base of agronomic research and routine laboratory data, enabling its application with limited cost. The OSI is a generic framework that can be adopted for specific regions with specific objectives. As a proof of concept, the OSI is implemented for all (>700,000) Dutch agricultural fields and illustrated with 11 pairs (“good” and “poor”) of local fields and 32 fields where soil quality and crop yield have been monitored. The OSI produced reasonable evaluation for most pairs when soil physical functions were refined with on-site soil visual assessment. The soil functions are sufficiently independent and yet together reflect complex multidimensionality of soil quality. The framework can facilitate designing sustainable soil management programs by bridging regional targets to field-level actions.

## Abstract 2

Soil health is important yet difficult to be properly evaluated. This study introduced a holistic soil assessment framework which is scalable, easily interpretable, transparent, and linked to farming practices.

## Introduction

To meet the increasing demands of a growing population, agriculture continues to intensify. Since the 1960s, world food production has increased drastically and agricultural production per capita has risen, while the use of machinery and fertilizer has also increased.^1^ Elevated inputs of nitrogen and phosphorus to agricultural soils have resulted in negative impacts on biodiversity, drinking and surface water quality, and human health.^2,3^ Both the agronomic potential of cropping systems and the environmental impacts of agriculture are strongly controlled by soil health. Soil health refers to the capacity of soil to function as a living ecosystem that sustains plants, animals, and humans and support ecosystem services including agricultural production.^4,5^ Healthy soils are not just a growing medium for crops, but they regulate and support essential ecosystem services, such as water purification, carbon sequestration, and nutrient cycling, and they provide habitats for biodiversity.^6,7^ Improving and sustaining soil health are therefore key to sustainable crop production. The term “soil health” is sometimes strictly distinguished from “soil quality”, with the former reflecting actual soil conditions (i.e., the condition of a given soil for a specific moment of time, which can deviate from achievable conditions of the soil due to, for example, past management) and the latter reflecting inherent soil conditions.^8^ In this paper, we follow this definition to refer to soil health.

To date, many attempts have been made to develop indices for assessing soil health,^6,7^ but an operational and reproducible methodology to assess soil health has not been developed so far.^9^ There is a broad consensus that multiple aspects of soils (e.g., chemistry, structure, and biology) and their interactions need to be considered. Further, various approaches have been proposed to translate soil attributes into indicators and aggregate them into an index, including (advanced) statistical methods and refined expert knowledge systems. Although these developments have rapidly increased the maturity of soil assessment methodologies, several challenges still remain. One of the major challenges is the lack of an explicit link between the soil assessment and the desired objective.^6,7^ Soil health is always assessed in relation to one or more objectives for which the soil is used, yet the relation between soil health and the objectives is rarely evaluated quantitatively and thereby, the interpretation schemes of the calculated indicator values are often unclear.^6^ This unclear link to objectives limits the adoption of the soil assessment tools by policy makers and practitioners. Another challenge is to effectively translate the soil assessment outcome to actionable information. Knowledge systems linking model outcomes to farming practices or socio-economic actions are often insufficient or inadequate, resulting in the lack of integrated soil assessment models to be used in decision making processes.^10^ The recently launched Soil Navigator is an example of an integrated assessment framework that incorporates multi-criteria decision models to provide management advice.^11^ However, their strong dependency on extensive classic soil analysis protocols and data from national monitoring programs hinders their application on a large scale. Last, to operationalize the soil assessment framework in various scenes, a framework must be scalable to different spatial and temporal scales. A scalable framework should be affordable (e.g., easy-to-obtain input data at low cost), adaptable to specific conditions of the assessment area, and expandable to new soil functions and objectives.

To overcome these challenges, we introduce a new soil assessment framework, the open soil index (OSI). The OSI builds on extensive soil and agronomic research to maintain a direct link to the objective (which is sustainable crop production), leverages routine laboratory data and public databases to make its large-scale application affordable, has a modular design to allow for easy adjustment and expansion, is developed in an open-source environment to assure transparency, and provides advice for field-level farming practices. In this way, OSI strives to provide an operational soil assessment that valorizes soil health and therewith promote sustainable soil management.

In this paper, we first elucidate the generic OSI framework, and then we show its implementation on Dutch agricultural fields with three case studies: a national application to generate large-scale overviews of soil health and two local applications to test its plausibility on the field level. In the first case study, the OSI was applied on all agricultural fields in the Netherlands (762,518 fields). We demonstrated how multiple soil properties are aggregated into soil health scores, how overall quality of agricultural soils is spatially distributed, what the major limitations in soil health are, and which farming practices have potential to improve soil health. We also investigated whether the chosen soil indicators cover a wide range of relevant soil functionality without much redundancy and to what extent the soil health scores are sensitive to deviation in soil properties and to aggregation algorithms. The second case study applied the OSI to 11 pairs of typical agricultural fields across the Netherlands, which each consisted of a “good” field and a “poor” field in terms agricultural performance. The third case study applied the OSI to 32 agricultural fields where crop yields and soil properties have been monitored over 4 years. Finally, we discuss the future perspectives of soil assessment frameworks for soil-regulated ecosystem services.

## Materials and Methods

### OSI Framework

#### Basic Principles of the OSI

The structure and philosophy of the OSI framework are highly influenced by the earlier multivariate soil assessment framework SMAF,^12,13^ which assesses soil health with three steps: indicator selection, indicator interpretation, and integration into an index. The OSI renewed this framework by embedding classic empirically underpinned agronomical insights. Soil health is evaluated in terms of the capacity to meet the specific objective of ecosystem services, in particular the sustainable production of food. More specifically, the soil should be able to maintain the current crop rotation and produce sufficient and healthy food for now and in the next decade. Sustainable means that the agricultural use of the soil should lead to minimal losses of nutrients to ground and surface water, which indirectly contributes to other ecosystem services, such as regulation of water quality.

The OSI has a hierarchical structure (see abstract art). The primary building block is “soil functions”. Soil functions quantify the role of soil in fulfilling and supporting the objectives. Some examples of soil functions for the objective “sustainable crop production” are nitrogen and water supply, soil aggregate stability, and disease suppressiveness. Each soil function is quantified based on measurable “soil properties”. Soil properties are characteristics of a soil that can be obtained from routine soil laboratory analyses from processed remote sensing data, visual observations (optional), and field properties derived from public data sets. Subsequently, the soil functions, which are expressed with its own unit, are scaled into unitless grades (“soil indicators”) that range between 0 (poor) and 1 (good), reflecting the distance to the desired optimum for that specific function. The soil indicators are further aggregated into an integral assessment score reflecting the weighted distance to target. Finally, recommendations are given for farming practices that can be implemented to improve soil health.

**Figure 1:**
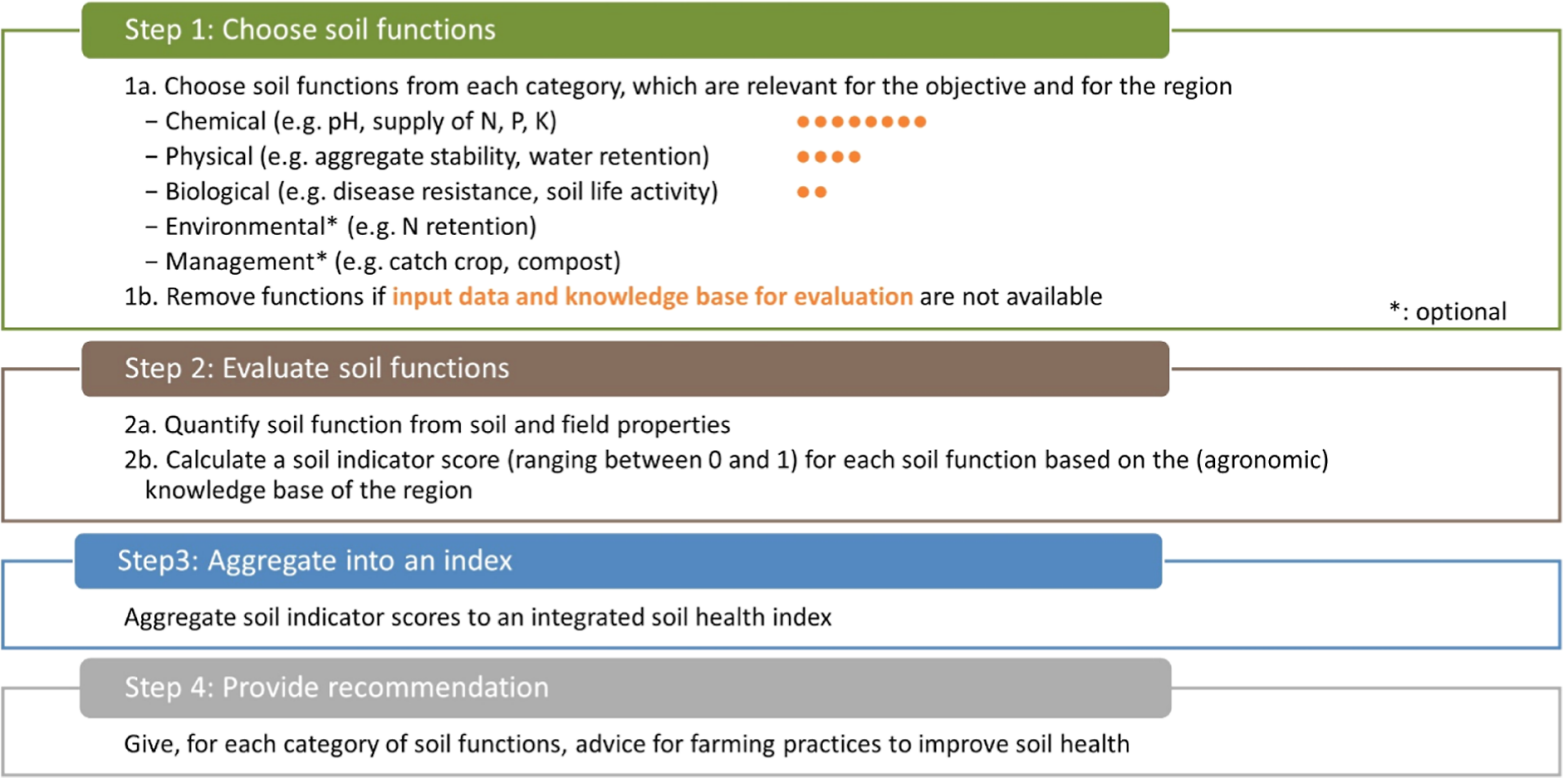
Four main steps to evaluate soil with the soil health assessment framework, the OSI. Orange points indicate the typical availability of input parameter data (from worldwide and national database, agronomic soil data) and the knowledge base for evaluation criteria for soil indicators of the three main categories.

The OSI is the basic framework that can be adopted for any specific region depending on the availability of data and the agronomic knowledge base (Figure 1). In the section below, each step is further elaborated in terms of its generic principles as well as a concrete example of implementation for Dutch agricultural fields. All algorithms, underlying assumptions, and references to original research for the Dutch case are published online and publicly available. The OSI has a modular structure, meaning that algorithms of soil functions and indicators can be easily modified with specific algorithms and threshold values based on new research results from national or international research programs. This enables a continuous and open international development of the OSI.

#### Soil Properties, Soil Functions, and Soil Indicators

Selecting the relevant soil functions to assess soil health is a crucial step. Following previous studies,^6,7^ the selection criteria of soil functions are that they, alone or in combination with others, (i) encompass various (e.g., chemical, biological, and physical) aspects of the soil, (ii) reflect variation in soil characteristics due to management but insensitive to day-to-day variation in weather, and (iii) play an important role in the region to achieve the objective. The relevant soil functions are subsequently selected based on the following criteria: whether they (i) can be quantified for individual fields based on public data (worldwide or national) or soil analyses in routine agronomic laboratories at minimum costs and (ii) can be evaluated based on the agronomic knowledge base applicable for the region concerned.

These soil functions are clustered around the five categories, namely, chemical, physical, biological, environmental, and management. The rationale of classifying into categories is that (1) it allows calculating sub-scores of each category before aggregating to an integral final score, which helps users to interpret the scores more easily, and (2) it enables users to exclude specific categories or set a heavier weight on specific categories. Data availability is typically very high for chemical soil functions, intermediate for physical soil functions, and low for biological soil functions, whereas the importance of all three aspects is well recognized. Furthermore, in most countries, agronomic knowledge is available to set threshold values for, at least, the main nutrients, pH, rootability, organic matter and microbial activity, or earthworm abundance. Therefore, we have set as a minimum requirement that each of these three aspects must include at least two functions.

For the Dutch implementation, 22 soil functions were selected. The chemical functions include the capacity of the soil to supply nitrogen, phosphorus, potassium, magnesium, copper, sulfur, and zinc as well as the capacity of the soil to buffer cations and the soil acidity. The physical functions are the aggregate stability, the crumbability, the capacity of soils to retain and supply water, the capacity to resist wind erosion, topsoil sealing, subsoil compaction, drought stress, and wetness stress. The biological functions are soil life activity (approximated by the potential mineralizable N pool) and disease resistance, whereas the environmental functions include the capacity of soils to minimize nitrogen loss to groundwater and surface water. The management assessment is derived from an expert-judgement evaluation system “Sustainable Soil Management label”.^14^ This label aggregates the information of soil management measures on a field into a score. The soil functions which are not included in the Dutch implementation of the OSI but could be crucial for other regions are heavy metal pollution, soil erosion, and pesticide retention. In particular, heavy metal contamination is an important risk factor in brownfield sites of many developing countries, although it never exceeded the critical threshold anymore in the Netherlands. When needed, heavy meal contamination should be incorporated in soil health assessment properly, considering metal speciation and bioavailability.^15^

The soil functions are quantified with soil and field properties based on agronomic research or model simulations. The soil and field properties consist of topsoil properties (routinely analyzed by agricultural labs), field properties retrieved from public data, and soil management properties (see Table S2). Topsoil properties include organic matter content, pH, soil mineralogy, intensity and capacity pools of nutrients, and biological assays like the potentially mineralizable nitrogen pool. Field properties are soil type, ground water level, and crop rotation (retrieved from formal registration by farmers for manure regulations). Soil management properties include intensity of specific crops in the rotation scheme (e.g., tuber crops, grassland), age of the grassland, drainage system, tillage system, use of catch crops, compost, lime, solid organic manure, and straw residues. The soil properties are recorded on a yearly basis, and multi-year records can be processed.

Soil functions, having their own unit, are scaled into a unitless grading system (“soil indicator”), in which the performance of the soil function is quantified as a numeric grade ranging between 0 (poor) and 1 (optimum). These indicator values reflect the “distance to target” (i.e., difference between the current and desired situations, while ensuring that other soil functions are not limiting), and the target values depend on, for example, soil type, land use, and geohydrology. The indicator value can be interpreted as good (>0.75), sufficient (0.5–0.75), and poor (<0.5). European countries have extensive knowledge bases on the quality of agricultural soils, usually embedded within Fertilizer Recommendation Guidelines,^16−18^ and Good Agricultural Practices.^19^ We used Dutch knowledge base^16,20^ to convert soil properties of each soil function to soil indicators. For nutrient-related soil functions, the target values were derived from field trials: indicator value 1 corresponds to the optimum level above which the yield does not respond, and indicator value 0.5 corresponds to the level under which (additional) fertilization is recommended. Additionally, some of the soil indicators are evaluated based on national monitoring networks as well as from (validated) simulation models. More details on each indicator are given in Supporting Information section A (Tables S1 and S3).

#### Visual Soil Assessments

Visual soil assessment (VSA) is a simple and rapid method to evaluate soil in situ by digging a hole and assessing several soil indicators visually. VSA can be a valuable and cheap addition to standard chemical and physical analysis to evaluate soil health. A study showed that four out of eight visual observations was well validated with standardized measurements, yet their correlation depends on soil types.^21^ The OSI implementation adopts the existing VSA system “BodemConditieScore”,^22^ a system widely adopted by farmers and extension services. It visually evaluates the following items with a qualitative score (poor/moderate/good) for the presence of earth worms, soil compaction, number of gray spots (indicative for waterlogged conditions), ponding, cracks, bio pores, rooting depth, soil structure, and crop cover. If VSA data are available, they replace the model-derived indicators for soil compaction and aggregate stability.

#### Integrating to Holistic Score

The indicator values of various soil functions can be aggregated into a single score that holistically represents the soil health. The common approaches to integrate soil functions into an overall score include equal-weight average, weighted average based on expert judgement, or data-driven approaches which typically build upon multivariate statistical modeling to link soil functions and objectives.^7^ The last approach is the most preferred, yet it requires a large data set including all relevant soil and field properties as well as quantified metrics of the soil objectives (e.g., crop yield or other ecosystem services), which are very scarce.

The OSI adopts a weighted averaging approach, with three aggregation steps (Figures S1): (1) the soil functions within each category (chemical, physical, biological, environmental, and management) are first aggregated on a yearly basis, then (2) the category sub-scores of multiple years are aggregated, and finally (3) the sub-scores of the five categories are aggregated into a final score. The advantage of the multiple aggregation steps is that it offers both integral assessment (i.e., whether a soil is overall good or not) and an assessment for each specific category or function individually (i.e., which soil functions are poorly evaluated and to what extent these soil functions need to be adapted to improve the overall soil health). Furthermore, by aggregating scores of multiple years with different soil properties and land use, the OSI can fuse the yearly evaluated actual capacity of a particular soil under the specific conditions (soil health^8^) and the inherent capacity of the soil (soil quality^8^).

Each aggregation step uses a correction factor to control the weights of each element. The correction factor of the first aggregation is computed based on the distance to target of each indicator. Built on the theory of von Liebig that crop yield is controlled by the most limiting resources, a poorly scoring soil indicator weighs more heavily. This way the lowest indicator, supposedly the most limiting factor for crop production, becomes more important than indicators being optimal already. The correction factor of the second aggregation is computed so that more weight is given to recent crops (<5 years) than previous (>5 year) crops. The correction factor of the third aggregation is based on the number of soil indicators that makes up the category. The rationale for giving a heavy weight for a category with more underlying indicators is that such a category, for example, chemical category, is better supported by measurable soil properties and better understood. The correction factor for aggregation can be adjusted by the user to reflect specific needs of the region. See Supporting Information B for a more detailed description of the correction factors.

#### Recommendation for Farming Practices

The OSI also provides recommendations for farming practices that improve the bottlenecks in soil health. For this purpose, a set of farming practices were selected and their impacts on each soil function were evaluated based on experimental evidence from the scientific literature. The selected farming practices include liming, compost application, no-till practices, use of tagetes or deep rooting crops, the use of catch crops, increasing grassland age, adjustment of fertilization, use of leguminous crops, improving botanical composition of grassland, and repairing subsoil compaction. For each category, the best practice was identified, which improves poorly scored soil functions most effectively. See Supporting Information A for more details.

### Case Studies

#### Case 1: Assessment of All Agricultural Fields in the Netherlands

To illustrate the potential of the OSI, the framework is applied for all agricultural fields in the Netherlands. Numerical soil properties were obtained for all fields using regression kriging models, which were built on covariables such as farm properties (animal numbers, fertilizer history), data from national monitoring networks (soil properties, atmospheric deposition), weather conditions, satellite data, and topography. The training and testing data sets of these geospatial models were derived from soil analyses of 110.000 fields measured between 2007 and 2017 by various agricultural laboratories.^23^ Categorical soil properties such as soil type and geohydrology were obtained by overlaying the fields with national maps. Management properties were estimated based on expert knowledge of typical practices for each soil type and land use type (grassland/maize land/arable land). These values do not reflect the actual practices on field levels, and therefore, the management score obtained in this exercise is not evaluated explicitly. Land use of the past 10 years (2010–2019) was retrieved from national registration, whereas the soil properties are assumed to be unchanged across 10 years. The soil health scores were calculated with the Dutch version of the OSI, open Bodem index, implemented with the calculator OBIC v.2.0.2.^24^

To explore general trends in soil properties, their variation among fields as well as differences between soil types and land uses were analyzed (see Supporting Information A). Relationships among soil indicators were tested with Spearman’s correlation test. Additionally, in order to extract the major axes of variation and therewith understand the relations between the indicators, principal component analysis (PCA) was conducted based on the correlation matrix of indicator values of 21 soil functions (i.e., excluding the soil function management). The final OSI score was computed through different calculation steps. Error propagation tests were conducted to estimate the sensitivity of the OSI score against a change in a single soil property and against different aggregation methods and how this depends on soil type, land use, and soil function category (see Supporting Information F).

#### Case 2: Paired Local Fields

In addition to the evaluation of the OSI on the national scale, we selected 11 pairs of local fields (*n* = 22) across the Netherlands. Each pair contains a “good” field and “poor” field owned by a farmer, located in the same region. The judgement of “good” or “poor” is based on the experience of the farmer regarding the soil status in terms of long-term crop yield (stability), with a criterion that the good field produces more than the paring poor field in the past decade. Note that since the yield data are not available for all fields and yield of different crops cannot be directly compared, the performance of the good and poor fields could not be objectively validated. For each field, a VSA was performed on site; management history was recorded; and required soil chemical, physical, and biological parameters were measured. Based on these data, the OSI was applied to evaluate the soil health. The indicator scores for the soil function “compaction” and “aggregate stability” were replaced by the VSA observations.

#### Case 3: Field Monitoring

From the ongoing monitoring network, we selected 32 fields across the Netherlands where both soil properties and crop yields have been collected from 2018 to 2021. We use this small dataset to illustrate the relationship between final soil quality score and crop yield.

## Results and Discussion

### Application of the OSI on Dutch Agricultural Fields

**Figure 2:**
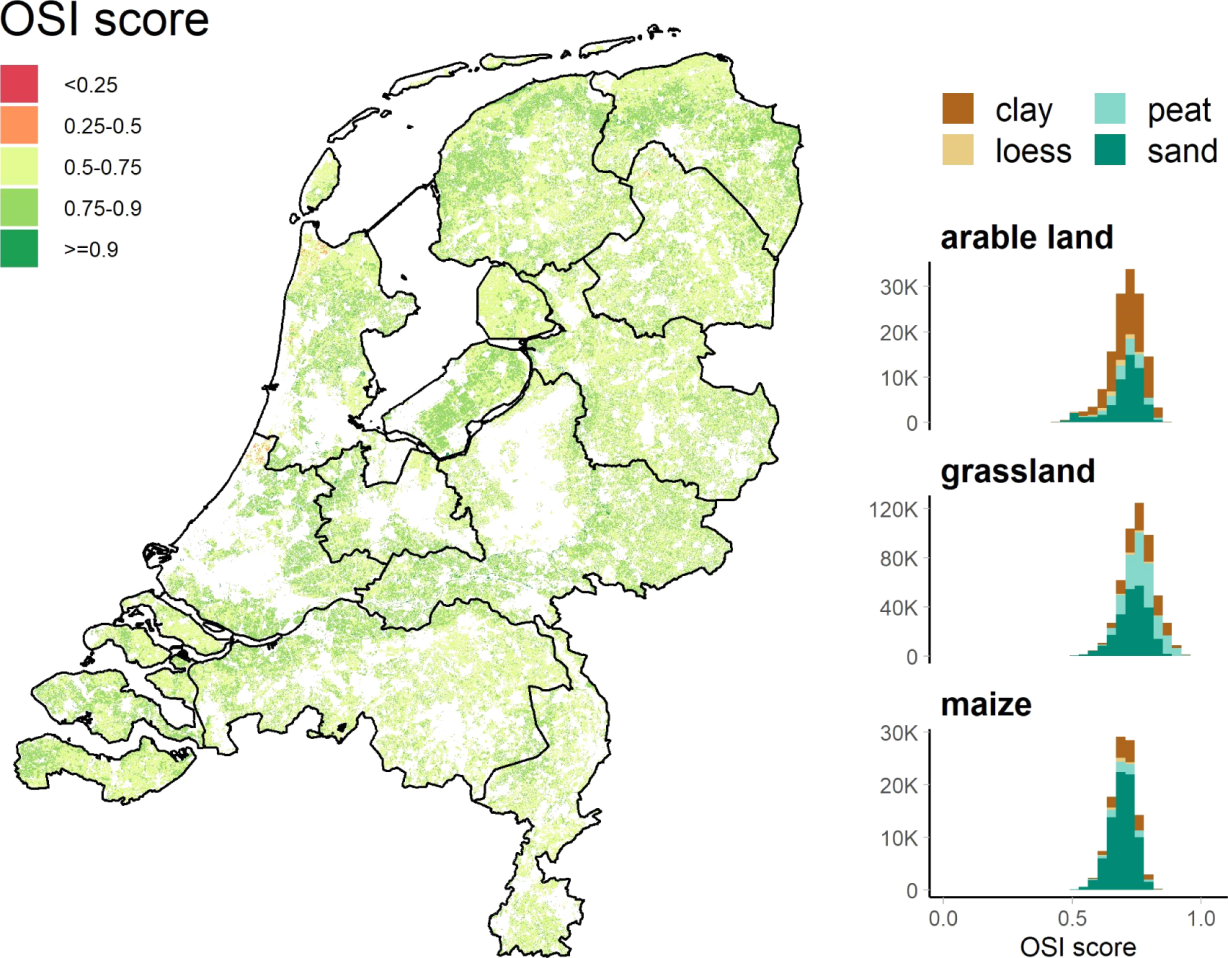
Spatial distribution of the final OSI score for all agricultural fields in the Netherlands (*N* = 762,518). Histograms are shown separately for different land uses. The score can be interpreted as follows: good (>0.75), sufficient (0.5–0.75), and poor (<0.5).

To illustrate the applicability of the OSI on a large scale, the OSI was applied to all agricultural fields of the Netherlands for the years 2010–2019. The overall soil health was sufficiently high (>0.5) for most fields (Figure 2). The mean value of the score was 0.73, and only 0.2% of fields scored less than 0.5. The final OSI scores were similar between crop types in terms of the mean value (0.72 for arable land, 0.73 for maize, and 0.74 for grassland) as well as the coefficient of variation of the final OSI score (ranging between 6 to 9%). Clay soils scored slightly higher (mean 0.75) than sand soils (mean 0.72). Nevertheless, the large differences observed in soil properties between soil types and crop types were less visible in the overall OSI scores since OSI evaluates the distance between the current and desired situations (and the latter varies depending on the soil type and land use). Among five sub-scores, the soil physical categories were often the weakest link of the overall soil health: the physical sub-score was the smallest among all categories for 34% of the fields, followed by 29% for the chemical sub-score and 27% for the environmental sub-score (Figure S4). When looking at individual indicator values of soil functions, 98% of fields had at least one soil indicator which scored poorly (<0.5). However, the majority of those fields scored poorly (score < 0.5) only for one indicator (18%), two indicators (33%), three indicators (26%), or four indicators (14%). Only 7% of fields had more than four poorly scored indicators. This also suggests that Dutch agricultural fields are in general well managed, with only a few crucial bottlenecks in terms of achieving sustainable crop production.

**Figure 3:**
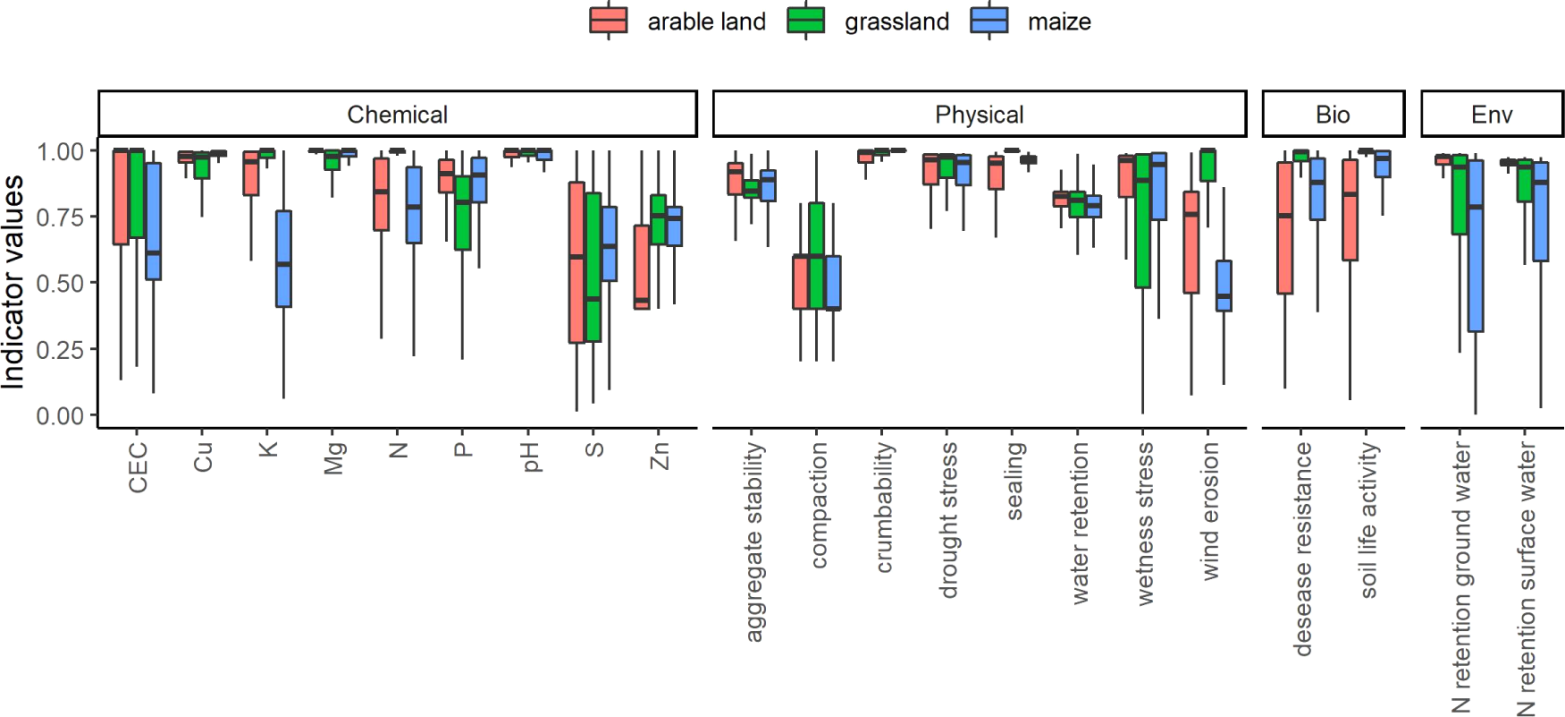
Indicator values of soil functions for Dutch agricultural fields (*N* = 762,518), shown separately for chemical, physical, biological, and environmental categories. The box depicts first-quantile, median, and third-quantile values, shown for different land uses separately.

To identify typical bottlenecks in Dutch agricultural fields, soil indicator values of each soil function were examined (Figure 3; also see Table S5). Of the eight physical indicators, soil compaction was most frequently evaluated as poor: 50% of fields scored less than 0.5. Furthermore, for 29% of fields, soil compaction had the worst score among all physical indicators, especially for arable fields on clay and loess soils. The indicator for vulnerability to wind erosion was also evaluated poorly: 24% of fields scored less than 0.5 and was the worst score among physical indicators for 14% of the fields, in particular for sandy arable fields. Most chemical indicators scored sufficiently high (>0.5) for the majority of fields, yet sulfur availability often scored poorly: 49% of fields scores less than 0.5. The score for sulfur availability was the lowest among the chemical indicators for 48% of fields across all crop types and soil types. Zinc, phosphor, and potassium availability were sometimes the bottleneck: 15% of fields, especially on clay soils, had the lowest score for zinc; 12% of fields, especially on peaty soils, had the lowest score for phosphor; and 6.6% of fields, mainly maize, had the lowest score for potassium. As for the two biological indicators, only a small portion of the field scored poorly (<0.5) for both disease resistance and soil life activity. As for two environmental indicators, 17 and 10% of fields scored less than 0.5 for N retention to groundwater and to surface water, respectively. N retention to groundwater scored often poor for maize fields.

**Figure 4:**
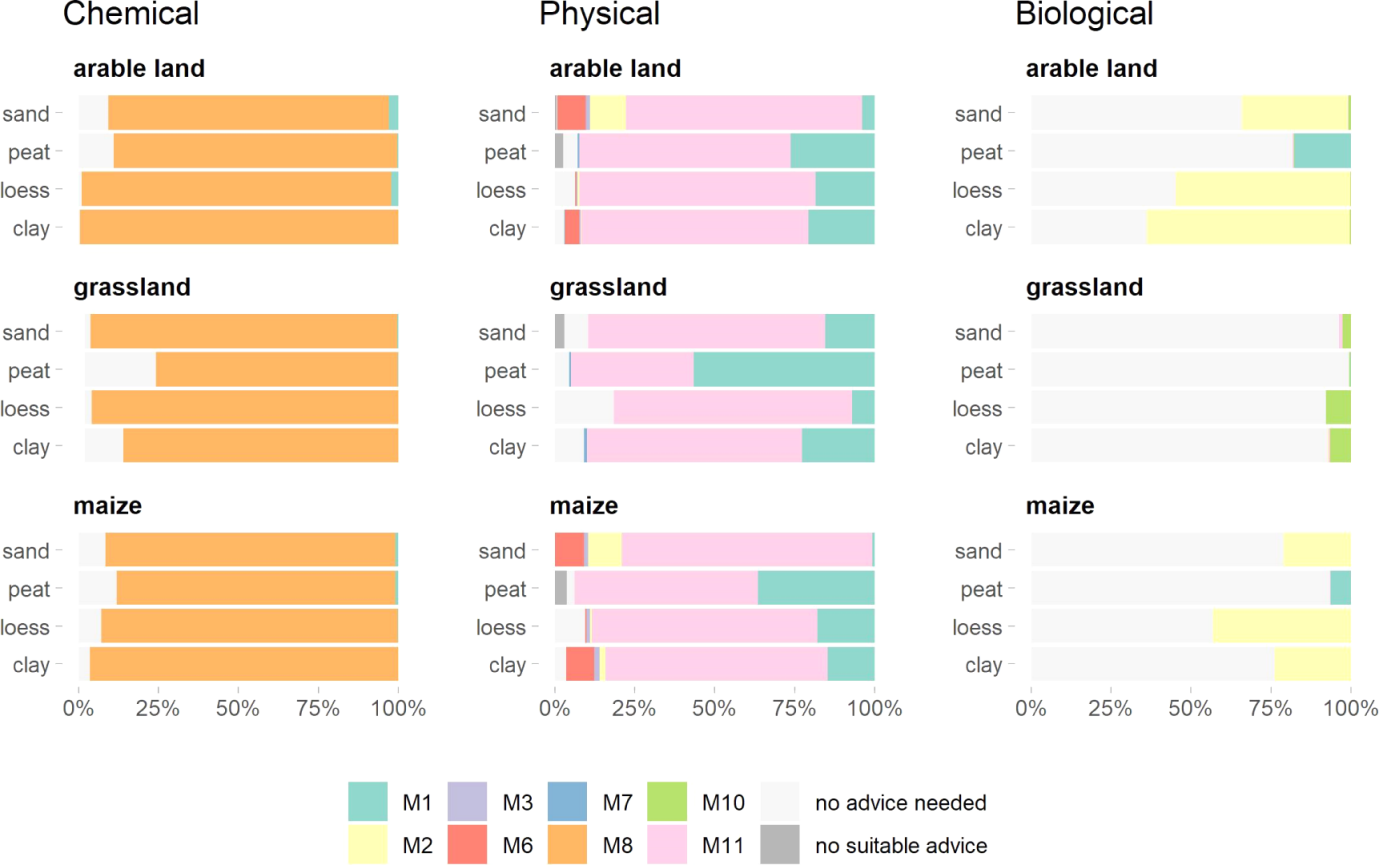
Recommended farming practices (chemical, physical, and biological measures) for Dutch agricultural field (*N* = 762,518). Results are shown separately for different land use and soil types. M1: liming, M2: compost application, M3: non-till practices, M6: use of catch crops, M7: increasing grassland age, M8: fertilization conform “maintenance and build-up approach”, M10: improve botanical composition of grassland, and M11: Repairing subsoil compaction.

Reflecting the poor score for soil compaction, the most recommended farming practice to improve the soil physical category was “subsurface compaction recovery (M11)” (Figure 4). Approximately 65% of fields received this as the most recommended practice. Measures aiming at improving soil organic matter, such as compost (M2) and green manure (M6), were also recommended for a small number of fields. To improve chemical category, “follow fertilization advice (M8)” was by far the most recommended practice, simply due to the fact that any nutrient deficiency can be solved by targeted nutrient applications. Liming (N1) was recommended to a small number of fields of arable lands, indicating that the pH in most soils was around the optimum value. Measures to improve biological soil functions were recommended for a minority of fields. Compost (M2) was the most frequently recommended practice for arable and maize fields, whereas some grasslands were recommended to improve botanical composition (M10).

### Application of the OSI on Field Scale

The OSI was applied to 11 pairs of agricultural fields, containing a “good” and a “poor” field (see Figure S10 for total scores, category sub-scores, and indicator values of individual soil functions for all fields). For 6 out of 11 pairs, the OSI final score was higher for good than for poor fields (Table 1). Chemical scores were sufficiently high for most fields (except for cases 11 and 12, which had low scores for S availability), indicating that chemical properties were hardly the distinguishing factor between good and poor fields. Compaction was often the most limiting soil function, for whole Dutch agricultural fields (see the first case study) but also for the 22 fields examined here. When the compaction was evaluated using the national data set with coarse resolution, the OSI failed to capture the local variation in compaction between good and poor as observed in the field with VSA (results not shown). Similarly, the OSI scores for aggregate stability, which is calculated based on SOM and CEC occupancy with Mg, Ca, and K, were not consistent with the aggregate stability evaluated with the VSA. When VSA scores were used to replace the OSI compaction scores and aggregate stability scores, the physical scores were high for the good field for 8 out of 11 fields. This indicates that on-site assessment for physical soil functions is desirable when small-scale comparison, rather than regional assessment, is the primary focus of the OSI application.

**Table 1:** Number of Pairs of Fields in Which the OSI Score is Higher for “Good” Fields Than “Poor” Fields (Good > Poor), Same (Good = Poor), or Higher for “Poor” Fields Than “Good” Fields (Good < Poor)^a^

type score	good > poor	good = poor	good < poor
OSI score	6	1	4
OSI sub-scores			
chemical	5	1	5
physical	8	0	3
biological	4	2	5
management	5	5	1
environmental	6	2	3
VSA	9	1	1

a The evaluation was made for the final OSI score as well as sub-scores of five categories. In addition, the evaluation for VSA score is also shown.

When applied on 32 fields with variable crop rotation schemes, there was a weak but positive relationship between the OSI score and the crop yield for potato, cereals, and sugar beets (Figure S14), thereby illustrating the relevance of soil health on crop performance. A more extensive monitoring is foreseen to assess the impact of soil health given the potential and confounding impacts of fertilizer, irrigation, and pest management.

### Redundancy and Error Propagation Analysis

Our sensitivity analysis provided several technical insights on the performance of the OSI. The correlation and multivariate analysis showed that the soil indicators were not strongly correlated and had enough levels of orthogonality (see Figures S5 and S6). Correlation among 21 soil indicators was weak to moderate, with only 9 pairs (out of total 210 pairs) having a positive correlation coefficient (ρ) higher than 0.5 and 6 pairs having a negative ρ lower than −0.5. The first eight axes of PCA accounted for 70% of the total variation, and there was no dominant component which explained a large portion of the variation: the first and second axes accounted for 20 and 13% of total variation, respectively. The loadings of the soil indicators spread over the PCA biplot of axis 1 and axis 2 without much overlap. These results indicate that the selected soil indicators are sufficiently independent on one hand and reflect the complex multidimensionality of the soil across land use and soil types on the other hand.

Error propagation analysis (see Supporting Information F) showed that no single soil property strongly influenced the final OSI score, even though certain soil attributes, such as soil organic matter, regulated various soil functions (Figure S7). Furthermore, the nonlinear weighing approach in the aggregation steps enabled to reflect a single poorly performing soil function in the final score (Figures S7 and S8). This is not achieved by evaluation systems in which a non-weighing or linear-weighing aggregation method is adopted.^25,26^ As pointed out in previous studies,^7^ aggregation is one of the elements in soil assessment frameworks that strongly influence the outcome. The consequence of aggregation methods therefore merits more careful consideration.

### Applicability of OSI for Other Regions

The OSI is a generic framework that can be adapted for specific regions, tuned for the available data and knowledge base of that region. Utilizing existing laboratory data has many merits as it facilitates large-scale application at low cost and analysis on temporal changes in soil health, and it helps easy communication with farmers who are familiar with the values and their implications. Soil data and the agronomic knowledge base are widely available worldwide: the registered national agricultural labs within the Global Soil Partnership of FAO cover the vast majority of the countries, their coverage is expanding all over the world, and most countries have protocols to measure and interpret soil parameters to generate fertilization recommendations (see Supporting Information J). Furthermore, the initiatives of harmonizing soil information have resulted in large-scale soil data sets, such as SoilGrids.^27^ Hence, a framework like OSI is worldwide applicable in most regions. When OSI is downsized to the most common 10 soil parameters (total N, available P, available K, CEC, pH, clay content, silt content, sand content, organic C, and bulk density), the Dutch fields were evaluated somewhat differently, yet the general trends could be sufficiently captured (Supporting Information K). This indicates that the use of more indicators can reflect wider dimensions of soil health and therefore is more comprehensive, but the downsized version of OSI could capture the essential part of the variability of soil health worldwide.

In this end, leveraging existing soil data and agronomic knowledge bases is preferred above the use of a minimum data set (MDS), the approach often adopted in soil assessment frameworks. Although a small number of soil attributes can produce a plausible soil assessment,^7^ the MDS approach is often limited since it involves specific measurements that are not available in routine labs and threshold values are often not universal. In an era of increasing data and knowledge availability, the efficient use of existing data in soil assessment can lower application thresholds.

### Proof of Concept

One of the expected roles of soil assessment tools is to quantify soil health on the large spatial scale and thereby contribute to decision making processes to achieve targets on regional and national levels. Our case study with all Dutch agricultural fields demonstrated a concrete example of how application of the OSI can be embedded in such an attempt. The outcome not only gives ideas for overall performance of agricultural soils and typical bottlenecks in soil functions but also produces maps of soil health on different levels, for example, final overall score and individual soil functions. In this way, the gradients of soil health can be related to other landscape elements, which helps local and regional governments to design tailor-made, area-specific strategies. Furthermore, hot spots of fields with high potentials and low potentials can be visualized, which can feed emerging debates on optimizing land use by redesigning configuration of agricultural lands to achieve multiple targets.^28^ The OSI also identifies recommended farming practices on field levels, which may be incorporated in the rewarding schema of governments or private organizations to stimulate sustainable soil management. Since anthropogenic impacts play a major role on soil health, such direct linkage of the soil assessment to local farming practices is indispensable to the promotion of sustainable agriculture, which constitute a part of the Sustainable Development Goals (SDGs) of United Nations.

One of the main challenges in soil assessment systems is the lack of solid validation studies. The soil functions of the OSI are evaluated based on well-established empirical evidence, and therefore, their individual scores are in theory verified, but the integrated final score requires validation. Our case study with 11 pairs of local fields and the assessment for 32 fields over a 4 year period provided a first proof that the OSI works on the field level. The soil health index matched the experience of the farmers for the majority of the cases, in particular when the soil physical properties were supplemented by the local visual assessment, and showed a positive response with crop yields. This highlights the potential of the OSI as well as the need for local data to refine soil parameters derived from national data sets. Our OSI scores of all Dutch agricultural fields was not quantitatively validated due to the absence of crop production data on the field level. In future, ideally, the national-level OSI scores should also be compared with measured ecosystem services, such as crop production and nutrient balance. Furthermore, the effects of the recommended farming practices, which are in principle already underpinned by empirical evidence from field experiments, need to be validated with long-term monitoring projects with farming practices. Although those validation data are difficult to obtain on a large scale, increasing availability of big data (such as those obtained from precision farming, satellite data, national monitoring network) will help close the gap. Since most of the existing data sets are designed for a specific purpose and therefore intrinsically one-dimensional, a key to successful validation is to build an integral monitoring system that can knit different temporal and spatial scales.

Another way to test the plausibility of the OSI is to compare the soil health scores with those from other soil assessment frameworks. A small additional exercise with our field data set revealed that the OSI yields similar scores as the well-known soil health index used in the USA (Supporting Information I), yet more elaborate comparison among different frameworks merits a separate study. Further, the advantages and disadvantages of the OSI compared to other frameworks are discussed (see Supporting Information H). One of the advantages of the OSI is its scalability. Earlier research showed that it is not trivial to upscale or downscale soil functions and management practices across different spatial scales.^29,30^ The OSI’s approach of leveraging existing agronomic evaluation systems (available in almost all countries) and routine soil laboratory data enables its application on a large spatial scale at low cost. Another advantageous feature is its direct and functional link to advice for farming practices. Since any global or national target in sustainable agriculture calls for local actions taken on field levels, the ability of a soil assessment tool to bridge between global targets and local actions is a prerequisite to be used in decision making processes.^8^ A good advisory tool for decision support should account for various motivations and standpoints of stakeholders.^31,32^ The proposed hierarchical modular structure built in an open-source environment allows farmers, policymakers, and advisors to adjust or replace functions to meet their own conditions and needs. A current disadvantage of the OSI is its relatively large number of required input parameters, which is easily attainable for countries like the Netherlands where the routine laboratory data are widely available, but not for others. However, due to the modular system, the input requirements can be adjusted to meet the data availability of the area of interest.

### Outlook

It is increasingly recognized that soil functions mediated by soil biota are crucial to maintain healthy soil,^6,33^ although the impact of soil biota on crop production is not yet fully understood. Recent advances in knowledge and data will help build more robust, process-based relationships between crop yield, soil biological properties, soil management, and disease resistance. For example, for plant parasitic nematodes, proper and affordable detection techniques and solid evaluation criteria are already established.^34^ As for generic soil biodiversity, no single metric can provide a full overview,^35^ and agricultural practices have inconsistent effects on soil microbial community.^36^ To include soil biodiversity in soil assessments, further empirical evidence and theoretical underpinning are needed on the interplay between agricultural practices, soil biota, and crop productivity.

The objective of the current OSI framework focuses on sustainable crop production, while in the context of SDGs, many other ecosystem services are recognized as crucial societal objectives that soil can contribute.^8^ Relevant soil ecosystem services from the SDGs include not only delivery of healthy food (SDG2 and SDG3) but also clean and sufficient water (SDG6), the mitigation of climate (SDG13), and the support for biodiversity and protection of land degradation (SDG15).^37^ Engaging farmers to consider these SDGs requires extension of the framework to include more environmental soil functions. The major challenge in including multiple objectives other than crop production is that quantitative research is lacking to underpin site-specific impacts of soil properties on these objectives. Furthermore, the trade-off between different objectives needs to be properly tackled as full synergies among many ecosystem services do not exist.^11^ The landscape approach to balance supply and demands for different services, such as “Functional Land Management Approach”,^29^ may provide new insights to better optimize soil-based ecosystem services beyond field and farm levels. To reach the multidimensional targets of the SDG, a paradigm shift is needed: the “wicked” problems cannot be solved with linear research approaches but require more stakeholder-oriented holistic approaches.^37^

## Supporting Information Available

A. Description of the soil functions and underlying properties of the Open Soil Index (Table S1–S3); B. Aggregation procedures of the OSI (Figure S1, Figure S2); C. Patterns of soil properties of Dutch agricultural fields (Figure S3, Table S4); D. Supplementary figures and tables of case study of Dutch agricultural fields (Figure S4, Table S5); E. Analysis on indicator complementarity and redundancy (Figure S5, Figure S6); F. Error propagation analysis (Figure S7, Figure S8, Figure S9); G. OSI test results for 22 field sites (Figure S10) and H. Comparison of soil assessment frameworks (Table S6); I. Application of other assessment frameworks (Figure S11,Table S7) ; J. Availability of soil data and agronomic knowledge base worldwide (Figure S12); K. OSI implementation with fewer indicators (Figure S13; Table S8) and L. OSI application on 32 fields (Figure S14). (PDF)
