# Supplementary material for: An Open Soil Health Assessment Framework Facilitating Sustainable Soil Management
Source: Environ Sci Technol. 2022 Nov 18;56(23):17375–84. doi: 10.1021/acs.est.2c04516 (PMC9730835; doi:10.1021/acs.est.2c04516)
Supplement: Supplementary file 1 — es2c04516_si_001.pdf [file es2c04516_si_001.pdf]

Supporting Information for:

An Open Soil health assessment framework facilitating sustainable soil management

Gerard H. Ros[1, 2*], Sven E. Verweij[1], Sander J.C. Janssen[3], Janjo De Haan[4] and Yuki Fujita[1]

[1] Nutrient Management Institute, Nieuwe Kanaal 7C, 6709 PA Wageningen, Netherlands

[2] Wageningen University and Research, Environmental System Analysis Group, PO Box 47, 6700AA Wageningen, the Netherlands

[3] Wageningen University and Research, Environmental Research, PO Box 47, 6700AA Wageningen, the Netherlands

[4] Wageningen University and Research, Plant Research, PO Box 16, 6700AA Wageningen, the Netherlands

* E-mail corresponding author: gerard.ros@wur.nl

Table of Content

A.	Description of the soil functions and underlying properties of the Open Soil Index	2
B.	Aggregation procedures of the OSI	12
C.	Patterns of soil properties of Dutch agricultural fields	14
D.	Supplementary figures and tables of study of Dutch agricultural fields	18
E.	Analysis on indicator complementarity and redundancy	20
F.	Error propagation analysis	22
G.	OSI test results for 22 field sites	26
H.	Comparison with other soil assessment frameworks	30
I.	Application of other assessment frameworks	34
J.	Availability of soil data and agronomic knowledge base worldwide	37
K.	OSI implementation with fewer indicators	39
L.	OSI application on 32 fields	41
Supporting References		42

Summary

Number of pages: 44

Number of figures: 14

Number of tables: 8

A. Description of the soil functions and underlying properties of the Open Soil Index

Each soil function incorporated in the Open Soil Index is quantified on the basis of several soil or field properties, and then evaluated into a unitless indicator score ranging between 0 (poor) and 1 (optimum). These indicator values reflect the 'distance to target' (i.e. difference between the current and optimum situation, while ensuring that other soil functions are not limiting). The further it deviates from score 1, the poorer the soil function works. For nutrient-related soil indicators, the optimum value is defined as the threshold value where crop yield is not responding anymore by addition of the nutrient. Indicator value 0.5 approximately corresponds to the threshold level of the fertilization guidelines under which (additional) fertilization is recommended (given the 'buildup and maintenance' approach as being implemented in fertilizer recommendation systems). Overall, the indicator value can be interpreted as good (>0.75), sufficient (0.5-0.75), and poor (<0.5).

For the Dutch implementation of the OSI, most of the target values were derived from the national and regional knowledge base embedded within Dutch fertilizer Recommendation Guidelines (CBAV, 2021; CBGV, 2018). The knowledge base is built on numerous amounts of classic empirical agronomic studies in the past decades. The optimum values in these studies were obtained from long-term fertilization experiment, fitting non-linear algorithms on field observations or from expert knowledge. Additionally, some of the soil indicators are evaluated based on national monitoring networks for soil and environmental quality as well from (validated) simulation models.

The evaluation criteria of each of the soil functions are described below. The underlying assumptions of the evaluation criteria are summarized in Table S1.

More details are available here: https://agrocares.github.io/Open-Bodem-Index-Calculator/

A.1. Soil chemistry and nutrient supply

The open soil index currently includes nine soil functions related to soil chemistry and nutrient supply. That are: the capacity of the soil to supply nitrogen, phosphorus, potassium, magnesium, copper, sulfur, and zinc, as well as the capacity of the soil to buffer cations and the soil pH.

Nitrogen supply

The nitrogen supply on grasslands is estimated based on the empirical relationship of N supply and total N level, which also varies depending on the sampling depth, soil texture and the age of the grassland (Hassink, 1995; Ros, 2011). For arable fields, the N supply is derived from a simple first order decomposition model, calibrated for Dutch circumstances and depending on soil texture, organic matter, and total N content (Janssen, 1984; Postma and Van Dijk, 2004; G H Ros and Bussink, 2013). The total N supply is evaluated via a parabolic scoring function with an optimum of 100 kg N ha^{-1} in arable fields and 140 kg N ha^{-1} in grassland fields.

Phosphor supply

The P availability for plants is strongly regulated by chemical dynamics of P between different pools. It is therefore important to consider not only directly available P pools but also P reserves in soil which supply soluble P to soil water. The phosphorus supply for grassland and maize fields is quantified based on soil P reserves (determined with ammonium lactate extraction) and plant available P (determined with CaCl2 extraction), where the optimum P supply is derived from multiple field experiments across the Netherlands (Ehlert et al., 2018; van Rotterdam et al., 2012, 2014). For arable fields, the phosphorus supply is approximated with water-extractable P (CBGV, 2018), which reflect P availability controlled by chemical sorption and desorption equilibria and affected by the iron and aluminum oxides content of soils as well as the availability of oxygen. The P supply is evaluated with the more-is-better logistic scoring function, which reaches the maximum score when the phosphorus supply is around 4.8 (unitless index, for maize and grassland fields) and 45 (mg P_2O_5 per liter, for arable fields) (van Rotterdam and Bussink, 2016).

Potassium supply

The potassium supply is strongly controlled by the distribution of K over different K pools in soil, which are regulated by sorption and desorption processes, the cation exchange capacity of the soil, and competing ions in soil solution and at the exchange complex. Building upon agronomic research done in last decades (Bussink et al., 2014; Den Boer et al., 2010; Ehlert et al., 1998; van Rotterdam, 2010), the K supply is quantified based on the plant available K fraction (measured via $CaCl_2$-extraction, K-$CaCl_2$) and the CEC for grasslands, K-$CaCl_2$ for maize fields, and K-$CaCl_2$, CEC, clay content, organic matter content and soil pH for arable fields. The K supply is evaluated with the more-is-better logistic scoring function.

Magnesium supply

The supply of Mg for arable fields as well as grassland fields on sandy soils is directly related to the plant available Mg-content (measured with $CaCl_2$-extraction). The Mg supply is subsequently evaluated with a more-is-better logistic response curve derived from the field experiments. For grassland fields on clay and peat soils, the Mg-supply is indirectly quantified based on Mg content in the grass (for intake by cows), which was predicted by a empirically-derived regression model of CEC, organic matter content, clay content, pH, as well as the K-availability assessed by K-$CaCl_2$ and K-CEC (Bussink DW, 1998; Den Boer DJ and Reijneveld JA, 1997; Ros and Bussink, 2011; Sluijsmans, 1987, 1967). Subsequently, the Mg supply is evaluated based on the difference between the predicted Mg and the optimum Mg content in grass.

Sulfur supply

The sulfur availability for grassland and maize is evaluated based on S supply from the soil, which was estimated from soil total S using empirical relationships (CBGV, 2018) . The S supply was subsequently evaluated with a more-is-better logistic scoring function, which reaches the maximum score at approximately 20 kg S/ha (CBGV, 2018). The availability of sulfur for arable land is evaluated based on the balance between the sulfur supply in the soil and the sulfur requirement of the crop (Oenema and Postma, 2003). The S supply in arable land is calculated using a simple first order decomposition model, which depends on soil organic matter, total S content, and soil texture (Janssen, 1984; Postma and Van Dijk, 2004; G H Ros and Bussink, 2013). S requirement of crops is estimated based on the agronomic region, land use, and soil type (CBAV, 2021). The difference between the S supply and S requirement is subsequently evaluated with a logistic scoring function, with which a more the requirement exceeds the supply, a lower the score is.

Copper supply

The supply and availability of Cu is evaluated based on Cu content in crops. Cu content in crop was estimated from the plant available Cu pool in soil (Cu-$CaCl_2$) for arable soils, and from the reactive Cu pool (Cu-HNO_3) for grassland soils, using empirical relationship (Bussink and de Haas, 2009). The Cu supply is evaluated with a more-is-better scoring function with an optimum value of 10 mg Cu kg^{-1} for grassland and 5 mg Cu kg^{-1} for arable land, derived from historical field experiments (Kabata-Pendias and Pendias, 2001).

Zinc supply

The supply of zinc is also evaluated based on Zn content in crops, which were estimated from the plant availability of Zn (measured with $CaCl_2$ extraction), pH and land use, using empirical relationships (Rietra et al., 2004). The Zn content in crop is evaluated with a more-is-better parabolic scoring function with an optimum of 100 mg Zn kg^{-1} (Kabata-Pendias and Pendias, 2001).

CEC

The cation exchange capacity determines the buffering capacity of soils to supply cations during crop growth. Soils with higher CEC values have a naturally higher soil fertility than soils with lower CEC values. The soil CEC is evaluated with a linear scoring function, which reaches the maximum score at a CEC value of 100 mmol+ kg^{-1} (Goselink and van Erp, 1999).

pH

The acidity of the soil is an important variable controlling the availability of nutrients as well as the activity in the rhizosphere. Liming recommendations have been derived from extensive field trials analyzing crop responses to varying pH levels in soil. Using these field experiments, the crop specific optimum pH levels can be determined given the pH, the crop rotation plan, the organic matter content of the soil and the soil texture (CBAV, 2021; CBGV, 2018) . The distance to the optimum pH is evaluated with a logistic evaluation function, with which the closer the actual pH is to the optimum pH, the higher the score is.

A.2. Soil structure and water availability

Soil physical functions include the aggregate stability, the crumbability of soil, the capacity of soils to retain and supply water, the capacity of soils to resist wind erosion, topsoil sealing, subsoil compaction, drought stress, and wetness stress.

Drought stress, wetness stress

The supply of water is of uttermost importance for crop development. The water supply depends on weather conditions and the capacity of soils to buffer and supply water during the growing season. Using long-term field experiments for multiple crops, relationships between soil texture, ground water levels, and yield depressions due to drought or too wet conditions were developed (Huinink, 2018; Van Bakel et al., 2005). Based on the relationships, yield suppressions due to wetness stress and drought stress, ranging from 0% (no damage due to stress) to 100 % (full damage on yield), are summarized in a table for each combination of groundwater level, soil type, and land use.

Water retention

Besides the response of the soil to extreme water conditions, the capacity of the soil to buffer water during the growing season is also important. The capacity to buffer water was approximated by plant available water (i.e. difference between water content at wilting point and field capacity) in topsoil. Plant available water was estimated with pedotransfer functions based on soil texture and organic matter content (Wösten et al., 2013, 2001, 1999), and was evaluated using a more-is-better logistic evaluation function.

Aggregate stability

Aggregate stability is evaluated given the cation occupation of the cation exchange capacity. Clay soils with 80% Ca, 8% Mg and about 3.5% K are recognized as soils with most stable aggregates due to the influence of electrostatic bindings of divalent cations (Bussink et al., 2008; Dontsova and Norton, 2002; Marshall and Holmes, 1979). Additionally, the crumbability of a soil, another measure for aggregate stability, was quantified based on clay content, the organic matter content and the pH (Huinink, 2018). The crumbability was then evaluated based on crop-specific potential risk on yield depressions.

Wind erosion

Wind erosion is highly controlled by the mineralogical composition as well as the presence of growing crops (Van Kerckhoven et al., 2007; Vroon HRJ, 2007). Using laboratory experiments from wind tunnels, an exponential function has been developed to estimate the erodibility risk for soils, given the clay and silt content of a field (G.H. Ros and Bussink, 2013). Grasslands are not susceptible for this risk.

Soil sealing

Sealing or crusting of soil happens when rain breaks down soil aggregate into particles, that creates slurry layers and forms a hard crust after drying. This hampers gas exchange of soil and hinders seed germination. The vulnerability of soils to sealing was quantified from the clay content and the organic matter content, based on field observations on yield depression in arable fields (Heeres and Van Erp, 1999; Huinink, 2018).

Compaction

Compaction is one of the serious threats to the crop production. The OSI uses the map of subsoil compaction risk, created with a soil compaction model SOCOMO for the whole Netherlands (van den Akker et al., 2012). Based on the national databases of soil and land use, the model determines whether the usual wheel loads for that land use exceed the strength of the subsurface under wet or moist conditions. Subsequently, soil properties and groundwater levels were used to determine whether the subsurface is particularly susceptible to compaction or whether natural recovery (by drought shrinkage, for example) is possible.

A.3. Soil biology and disease suppressiveness

There are a variety of ways that biological activity is measured in soils. Few, if any, of these measures are used routinely to test soils. The various methods of assessing soil biological activity differ in their accuracy, difficulty, and suitability for use. Currently, the most relevant include microbial activity, disease resistance, fungal-bacterial ratios, nematode and earthworm densities. In the current OSI, microbial activity and disease resistance are included.

Soil life activity

A common indicator to assess soil microbial activity is potentially mineralizable nitrogen (PMN). PMN is the capacity of soil microbes to mineralize organic nitrogen into plant available form of nitrogen, and usually measured with anaerobic or aerobic incubation methods. The potential microbial activity is evaluated using a more-is-better logistic curve with an optimum PMN value around 45 mg N kg^{-1}, following the scoring function of the CASH model (Moebius-Clune et al., 2016) .

Disease resistance

The capacity of soils to regulate soil borne plant pathogens, or disease suppressiveness, is key to soil quality (Bünemann et al., 2018). The disease suppressiveness has been related to various chemical, physical and biological soil parameters. However, correlations between soil suppressiveness and these soil parameters are not always consistent, depending on the pathogen and the system studied, which limit the possibility to apply an empirical multivariate model to predict disease suppressiveness in a soil assessment framework. Alternatively, Hanegraaf et al. (2013) proposed to use the soil organic matter content as an early detector for general disease suppressiveness based on expert-based algorithms. Organic-rich soils favor disease-suppressing microbes by providing food and by improving soil structure and moisture conditions. Using a more-is-better logistic scoring function, disease suppressiveness is assessed based on the soil organic matter content with an optimum value around 5%.

A.4. Environmental aspects

Sustainable crop production is inevitably linked to reduced impacts on environment, since efficient use of resources leads to minimizing negative impacts on environment. In the current version of the OSI, two soil functions of environmental categories are included: N retention for groundwater and N retention for surface water. This refers to the nutrient buffering capacity of soil that controls the risk of nitrogen losses to groundwater and surface water.

N retention for groundwater

The Dutch manure legislation uses the process-based models to quantify risks of N loss (Groenendijk et al., 2016). The input parameters for the model include the soil organic matter content, the decomposability of the organic matter, soil texture and groundwater dynamics, and the model has been calibrated with empirical dataset. The OSI uses the simulation output of this model to estimate fraction of net N surplus which is leached to groundwater. The fraction was calculated per soil type, land use, and groundwater level. N leaching to groundwater was estimated by multiplying the net N surplus (which is calculated based on soil nitrogen supply and N recovery rate) and the fraction of net N surplus which is lost to groundwater. The calculated N leaching to groundwater is evaluated given a logistic evaluation curve aiming to minimize any N loss due to leaching or surface runoff.

N retention for surface water

N retention to surface water was estimated in the same manner as N retention to groundwater, but using the fraction of net N surplus which is lost to surface water (via routes of runoff, drainage and shallow leaching).

A.5. Soil management

Unlike other soil functions, soil management is hard to evaluate quantitatively. To address this challenge, the OSI adopts the expert-judgement driven framework of the Sustainable Soil Management label (Van Der Wal et al., 2016). This label, designed by a group of soil scientists and consultants in the Netherlands, aims at stimulating sustainable soil management by means of transparent and systematic protocol to evaluate measures on field scale. In the framework, a number of soil management measures were selected that positively contribute to the ecosystem functions of the soil, and their impact on the soil quality was evaluated with points. The sum of all points of applied measures on the field was then used to calculate the score of management.

A.6. Soil and field properties

Table S2 describes the soil and field properties used as input for the OSI. Table S3 summarizes which soil properties were used as input to calculate each soil indicators.

A.7. Recommended farming practices

Eleven farming practices were selected for the recommendations to improve soil quality . The selected farming practices are: liming (M1), compost application (M2), no-till practices (M3), use of Tagetes in crop rotation scheme (M4), use of deep rooting crops in crop rotation scheme (M5), the use of catch crops (M6), increasing grassland age (M7), fertilization conform "maintenance and build-up approach" (M8), the use of leguminous crops (M9), improving botanical composition of grassland (M10), and repairing subsoil compaction (M11). Based on literature, the expected impacts of the farming practices on all soil functions of 3 categories

(chemical, physical, and biological) were quantified with an ordinal scale (very positive/positive/slightly positive/little effect/slightly negative/negative). The impacts were evaluated for three land use and four soil types separately.

The database of the impacts are saved as “recom_obic.RData” on github:

https://github.com/AgroCares/Open-Bodem-Index-Calculator/blob/v2.0.2/data/

For each category, the best practice was identified that improves poorly scored soil functions most effectively. When the indicator value of a soil function is high (i.e. > 0.6 for soil functions of chemical category and > 0.7 for soil functions of physical and biological categories), then we consider that no farming practice is particularly needed to improve that soil function and therefore the impacts of farming practices on the soil function were not considered. The effects of each farming practice on all soil indicators above the threshold value are summed up, and the farming practice which has the highest sum is chosen as the best practice. When all indicator values are above these threshold values, then no advice was given. When no farming practice can improve any of the poorly-scored soil function, then no suitable advice can be given.

A.8. Open Soil Index Algorithms and documentations

For the two case studies in the Netherlands, The OSI scores were calculated with the Dutch version of the OSI, Open Bodem Index, implemented with the calculator OBIC v.2.0.2 (Verweij et al., 2022). All algorithms are available on github via:

https://github.com/AgroCares/Open-Bodem-Index-Calculator

List of all functions and supporting vignettes can be found via:

https://agrocares.github.io/Open-Bodem-Index-Calculator/

More details of each soil function, such as underlying assumptions and references to original research are summarized as a Factsheet (currently only available in Dutch) and published online (www.openbodemindex.nl).

Table S1. Summary of the underlying assumptions of evaluation criteria of each soil functions

OSI function	Response type	Optimum value	Evidence type	References
Chemical				
Nitrogen supply	More is better, parabolic (till optimum)	100 kg N-NLV ha^{-1} (arable land), 140 kg N-NLV ha^{-1} (grassland)	Frequently observed values of existing Dutch dataset	(NMI, n.d.)
Phosphor supply	More is better, logistic	4.8 P-index (grassland), 45 mg P_2O_5 / L (arable fields)	Field fertilization experiment on 58 fields over 2 years, plus field observation of 144 fields	(CBAV, 2021; CBGV, 2018; van Rotterdam and Bussink, 2016)
Potassium supply	More is better, logistic	3.5 K-index (grassland), 25 K-index (arable land)	2-year fertilization experiment (for grassland); Large (>7000) database of Dutch agricultural soil (for arable land)	(Bussink et al., 2014; CBGV, 2018; Ros and Bussink, 2011)
Magnesium supply	More is better, logistic	132 Mg-index (grassland), 75 Mg-index (arable land), 105 Mg-index (maize)	Field fertilization experiment (N=70)	(Bussink DW, 1998; CBAV, 2021; CBGV, 2018; Sluijsmans, 1987, 1967)
Sulfur supply	More is better, logistic	20-23 S-index (grassland and maize), 0 S-balance (arable land)	Field fertilization experiments (grassland); balance between (modelled) S supply and crop- and region-specific S requirement by crop (arable land)	(CBAV, 2021; CBGV, 2018; Oenema and Postma, 2003)
Copper supply	More is better, logistic (grassland) or parabolic (till optimum) (arable land)	10 mg Cu kg^{-1} (grassland), 5 mg Cu kg^{-1} (arable land)	Field experiment	(CBAV, 2021; CBGV, 2018; Kabata-Pendias and Pendias, 2001)
Zinc supply	More is better, parabolic (till optimum)	100 mg Zn kg^{-1}	Literature study	(Kabata-Pendias and Pendias, 2001)
CEC	More is better, linear (till optimum)	100 mmol+ kg^{-1}	Literature study	(Goselink and van Erp, 1999)
pH	Less is better (in terms of the distance between optimum and actual pH), logistic	4.2 - 7.2, depending on crop type and soil type	Field liming experiments	(CBAV, 2021; CBGV, 2018)
Physical				
Drought stress, wetness stress	Less is better (in terms of yield suppression), linear	0% yield suppression	Soil-water-crop model, fed by long-term field experiments	(Huinink, 2018; Van Bakel et al., 2005)
Water retention	More is better, logistic	100 mm water in soil of 0-30 cm depth	Pedotransfer function of available water, based on Dutch soil database	(Wösten et al., 2013, 2001, 1999)
Aggregate ability	Less is better (in terms of deviation from optimum cation occupations), linear	no deviation from optimum occupations: 80% Ca, 8% Mg, 3.5% K	Literature study	(Bussink et al., 2008; Dontsova and Norton, 2002; Marshall and Holmes, 1979)
Crumbability	More is better, linear	10 crumbability-index	Field observations	(Huinink, 2018)
Wind erosion	Less is better, linear	0 wind-erosion-index	Laboratory experiment with wind tunnels	(G.H. Ros and Bussink, 2013)
Soil sealing	More is better, logistic	10 soil-sealing-index	Field observation	(Heeres and Van Erp, 1999; Huinink, 2018)
Compaction	Less is better, categorical (5 classes)	Risk class 1	Compaction model	(van den Akker et al., 2012)
Biological				
Soil life activity	More is better, logistic	45 mg N kg^{-1} $week^{-1}$	Literature study	(Moebius-Clune et al., 2016)
Disease resistance	More is better, logistic	4-10 % soil organic matter	Expert judgement	Hanegraaf et al. (2013)
Environmental				
N retention groundwater	Less is better, logistic	0 mg NO_3 L^{-1} extra leaching	Simple model fed by N model output and field properties	(Groenendijk et al., 2016)
N retention surface water	Less is better, logistic	0 mg N L^{-1} extra run-off	Simple model fed by N model output and field properties	(Groenendijk et al., 2016)
Management				
Management	More is better, linear	40 points	Expert judgements	(Van Der Wal et al., 2016)

Table S 2. List of soil properties used in the Open Soil Index. The source of the data is D: routine laboratory analysis data (extrapolated to all fields with geostatistical model), P: public data, M: validated model, S: processed satellite data, U: User defined, V: visual assessment on site.

variable	description	Unit / categories	Source
A_SOM_LOI	Organic matter	%	D
A_SAND_MI	Sand content	%	D
A_SILT_MI	Silt content	%	D
A_CLAY_MI	Clay content	%	D
A_PH_CC	Soil acidity	-	D
A_N_RT	Total N content	mg N/kg	D
A_CN_FR	Soil C:N ratio	ratio	D
A_S_RT	S soil reserves	mg S/kg	D
A_N_PMN	Microbial activity	mg N/ kg	D
A_P_AL	P soil reserves (ammonium lactate extractable)	mg P2O5/100 g	D
A_P_CC	P plant available (CaCl2 extractable)	mg P/kg	D
A_P_WA	P water (water extractable)	mg P2O5/ L	D
A_CEC_CO	Cation exchange capacity	mmol+/kg	D
A_CA_CO_PO	Ca occupancy CEC	%	D
A_MG_CO_PO	Mg occupancy CEC	%	D
A_K_CO_PO	K occupancy CEC	%	D
A_K_CC	K plant available	mg K /kg	D
A_MG_CC	Mg plant available	mg Mg/kg	D
A_MN_CC	Mn plant available	ug Mn/ kg	D
A_CU_CC	Cu plant available	ug Cu/ kg	D
A_ZN_CC	Zn plant available	ug Zn/ kg	D
B_GWL_CLASS	Groundwater level	10 categories (shallow to deep groundwater level, depending on soil groundwater depth in summer and winter)	P
B_SOILTYPE_AGR	Agronomical soil type	9 categories (e.g. river clay, marine clay, wind deposited sand, loam, and peat, peaty clay)	P
B_LU_BRP	Crop type	284 categories (e.g. consumption potato, permanent grassland, maize)	P
B_AER_CBS	Key agricultural area	14 categories	
B_SC_WENR	Risk of soil compaction	10 categories (‘very limited risk’ to ‘very large risk’)	M
B_HELP_WENR	Soil type for water stress evaluation	2233 categories (each category is associated with a drought and a wetness stress index of 0-100, depending on their land use and groundwater level)	M
M_LIME *	Has field been limed in last three years	Yes / No	U
M_GREEN *	Use of green manure	Yes / No	U
M_COMPOST *	Frequency of compost application	Yes / No	U
M_NONBARE *	Field 80% of the year green	Yes / No	U
M_EARLYCROP *	Use of early crops	Yes / No	U
M_SLEEPHOSE *	Manure application using sleep hoses	Yes / No	U
M_DRAIN *	Under water drains available	Yes / No	U
M_DITCH *	Ditch is cleaned and sludge is applied	Yes / No	U
M_UNDERSEED *	Underseed of grass at maize	Yes / No	U
M_NONINVTILL *	Non inversion tillage	Yes / No	U
M_SSPM *	Soil Structure Protection Measures	Yes / No	U

M_SOLIDMANURE *	Use of solid manure	Yes / No	U
M_STRAWRESIDUE *	Application of straw residues	Yes / No	U
M_MECHWEEDS *	Use of mechanical weed protection	Yes / No	U
M_PESTICIDES_DST *	Use of DST for pesticides	Yes / No	U
A_EW_BCS *	Presence of earth worms	poor / moderate / good	V
A_SC_BCS *	Presence of compaction of subsoil	poor / moderate / good	V
A_GS_BCS *	Presence of waterlogged conditions, grey spots	poor / moderate / good	V
A_P_BCS *	Presence / occurrence of water puddles on the land, ponding	poor / moderate / good	V
A_C_BCS *	Presence of visible cracks in the top layer	poor / moderate / good	V
A_RT_BCS *	Presence of visible tracks / rutting or trampling on the land	poor / moderate / good	V
A_RD_BCS *	Rooting depth	poor / moderate / good	V
A_SS_BCS *	Soil structure	poor / moderate / good	V
A_CC_BCS *	Crop cover on the surface	poor / moderate / good	V

**: optional*

Table S 3. List of soil indicators and the soil properties with which the indicator is calculated from. [C]: formula to calculate the indicator differs among crop types ('B_LU_BRP'), [S]: formula to calculate the indicator differs among soil type ('B_SOILTYPE_AGR')

Indicator	Description	Input soil properties
Chemical		
I_C_CEC	Cation Exchange Capacity	A_CEC_CO
I_C_CU	Copper availability	A_SOM_LOI, A_CLAY_MI, A_K_CC, A_MN_CC, A_CU_CC, [C]
I_C_K	Potassium availability	A_SOM_LOI, A_CLAY_MI, A_PH_CC, A_CEC_CO, A_K_CO_PO, A_K_CC, [S], [C]
I_C_MG	Magnesium availability	A_SOM_LOI, A_CLAY_MI, A_PH_CC, A_CEC_CO, A_K_CO_PO, A_MG_CC, A_K_CC, [S], [C]
I_C_N	Nitrogen availability	A_N_RT, A_CN_FR, A_SOM_LOI, [S], [C]
I_C_P	Phosphorus availability	A_P_AL, A_P_CC, A_P_WA, [C]
I_C_PH	Soil acidity	A_SOM_LOI, A_CLAY_MI, A_PH_CC, [S], [C]
I_C_S	Sulphur availability	A_SOM_LOI, A_S_RT, B_AER_CBS, [S], [C]
I_C_ZN	Zinc availability	A_PH_CC, A_ZN_CC, [S], [C]
Physical		
I_P_CEC	Aggregate stability	A_SOM_LOI, A_K_CO_PO, A_CA_CO_PO, A_MG_CO_PO, [S] [*1]
I_P_CO	Compaction	B_SC_WENR [*1]
I_P_CR	Crumbability	A_SOM_LOI, A_CLAY_MI, A_PH_CC, [C]
I_P_DS	Drougthstress	B_HELP_WENR, B_GWL_CLASS, [C]
I_P_WS	Wetnessstress	B_HELP_WENR, B_GWL_CLASS, [C]
I_P_DU	Wind erosion	A_CLAY_MI, A_SILT_MI, [C]
I_P_SE	Soil sealing	A_SOM_LOI, A_CLAY_MI
I_P_WRI	Water retention	A_CLAY_MI, A_SAND_MI, A_SILT_MI, A_SOM_LOI
Biological		
I_B_DI	Disease / pest resistance	A_SOM_LOI
I_B_SF	Soil life activity	A_N_PMN, [S], [C]
Environmental		
I_E_NSW	Nitrogen retention for surface water	A_N_RT, A_CN_FR, A_SOM_LOI, B_GWL_CLASS, B_AER_CBS, [S], [C]
I_E_NGW	Nitrogen retention for ground water	A_N_RT, A_CN_FR, A_SOM_LOI, B_GWL_CLASS, B_AER_CBS, [S], [C]
Management		
I_M	Soil management evaluation	A_SOM_LOI, A_P_AL, A_P_WA, B_GWL_CLASS, [S], [C], M_COMPOST, M_GREEN, M_NONBARE, M_EARLYCROP, M_SLEEPHOSE, M_DRAIN, M_DITCH, M_UNDERSEED, M_LIME, M_NONINVTILL, M_SSPM, M_SOLIDMANURE, M_STRAWRESIDUE, M_MECHWEEDS, M_PESTICIDES_DST

[*1]: Optionally, this indicator can be calculated based on the scores of Visual Soil Assessment (VSA)

B. Aggregation procedures of the OSI

The OSI includes three aggregation steps to calculate the final score (Figure S 1).

The first aggregation step combines the soil indicators within each category (chemical, physical, biological, environmental, and management) and compute a sub-score of each category of the year. The weighing score on soil indicator values is introduced so that low scores weigh more heavily (Figure S 2 left). The weighing score of soil indicator *i*, vcf_i, is calculated as follows:

$$vcf_i = \frac{1}{I_i + 0.2} \qquad \text{Eq. (B.1)}$$

where I_i is the value of the soil indicator for indicator *i*.

Subsequently, the sub-score of a category is computed, for each year *y*, by aggregating weighted values of all soil indicators within the category:

$$SS_{y,a} = \Sigma_i \left(I_i \cdot \frac{vcf_i}{\Sigma_i vcf_i} \right) \qquad \text{Eq. (B.2)}$$

where $SS_{y,a}$ is the sub-score of the category *a* for the year *y*.

The second aggregation step combines the sub-scores of multiple years to compute a single sub-score per location without temporal dimension. The weighing factor is computed so that more weight is given to recent years (Figure S 2 middle). The weighing factor for the year aggregation is formulated as:

$$ycf_y = ln(12 - min(y, 10)) \qquad \text{Eq. (B.3)}$$

where y is the length of years before the assessment. Y =1 means the year for which the assessment is conducted for (i.e. the most recent year), y = 5 means 5 year before the assessment. The maximum limit of the year was set to 10 years, thus the records older than 10 years are weighed as much as 10 year old record.

Subsequently, the soil sub-scores of multiple years are aggregated as follows:

$$SS_a = \Sigma_y \left(SS_{y,a} \frac{ycf_y}{\Sigma_y ycf_y} \right) \qquad \text{Eq. (B.4)}$$

where SS_a is the sub-score of the category *a*, without time dimension.

The third aggregation step combines five sub-scores into a final OSI score. The correction factor is based on the number of the soil indicators that make up the category. The more indicators the category include, the relatively higher weight the category is assigned (Figure S 2 right). The rationale is that the category with more underlying indicators, such as chemical

category, is better supported by measurable soil properties and better understood. The weighing factor is formulated as:

$$ccf_a = ln(ncat_a + 1) \qquad \text{Eq. (B.5)}$$

where $ncat_a$ is the the number of soil indicators included in the category *a*.

The final score is then calculated as:

$$S = \sum_a \left(SS_a \frac{ccf_a}{\sum_a ccf_a} \right) \qquad \text{Eq. (B.6)}$$

where S is the final OSI score.

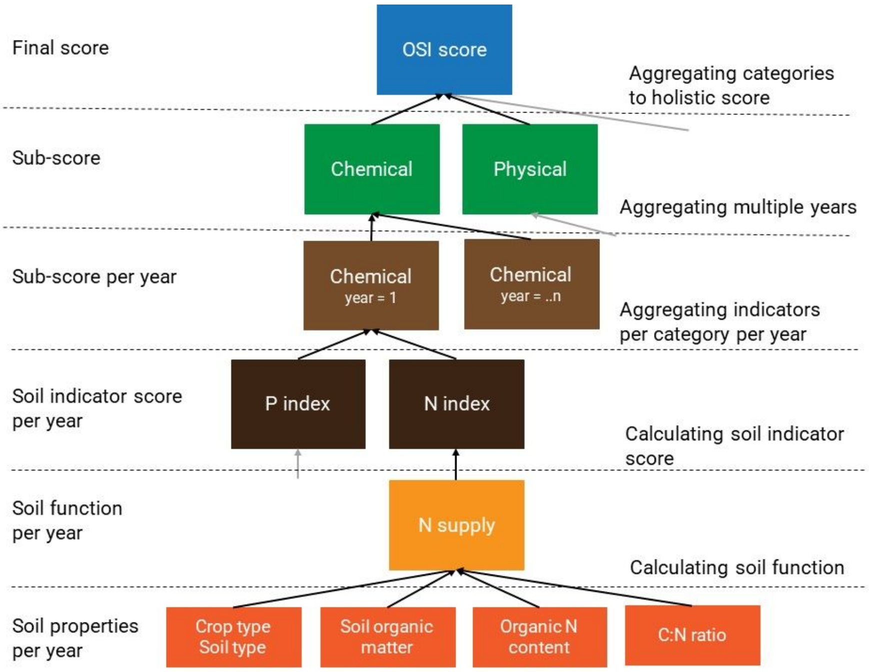

Figure S 1. Diagram of aggregation steps of the Open Soil Index

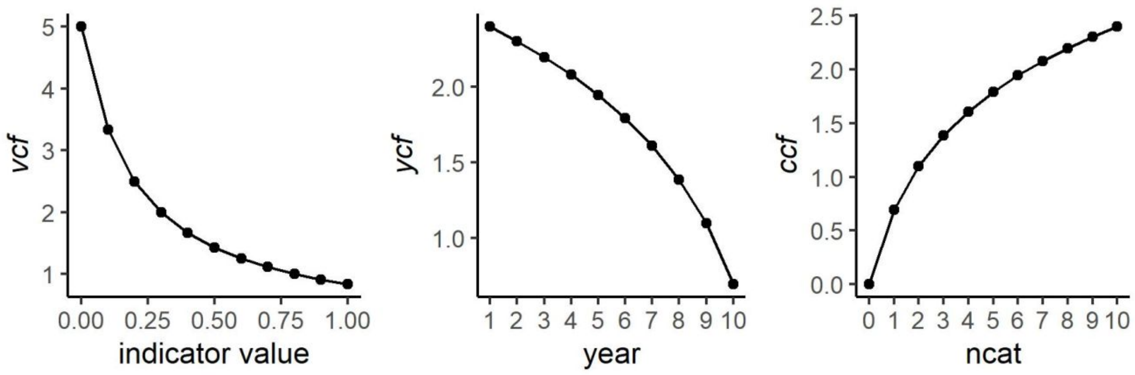

Figure S 2. Weighing factor of three aggregation steps: aggregation of indicator values within each category (left), aggregation of multiple years (middle), and aggregation of category sub-scores into a holistic OSI score (right).

C. Patterns of soil properties of Dutch agricultural fields

As input values for the OSI framework, a number of soil properties were collected for all fields of Dutch agricultural soils. Here we conducted several analyses to explore the general trends of the soil properties in the Netherlands.

Methods

For the numerical soil properties, their variations among fields were tested with quartile coefficient of dispersion (QCD). QCD is the non-parametric analogue to coefficient of variation, and is calculated by dividing the interquartile range (i.e. difference between the 75th and 25th percentiles) by the median values.

The effects of soil type and land use on soil property values were tested with ANOVA. To prevent extreme values from obscuring the general trends, only values between the 5th and the 95th percentile were included in ANOVA. Prior to the analysis, right-skewed variables (skewness greater than 1) were log-transformed. The residuals of ANOVA models were unimodal and approximately symmetrically distributed for all soil properties, indicating that the assumption of ANOVA test is not severely violated. Due to the large sample size, the test detects that the effects are always significant (Type-I error). Therefore, we evaluate the effect of soil type and land use by eta square values, instead of p-values of the coefficients. Eta-square is a measure of effect size, defined as the proportion of variance accounted for by the effect of interest over the total variance.

Results

General trends

The distribution of numerical soil properties of all Dutch agricultural soils is shown in Figure S 3. Median clay content values ranged from 4.5% in sandy soils up to 29% in clay soils. Organic matter increased from on average 3.3% in arable silty soils up to 13% in peaty grassland soils. The median pH values ranged between 5.1 up to 7.0 whereas the median CEC values ranged from 66 up to 363 mmol+ kg^{-1}. Most of the CEC was occupied by calcium (varying from 67% up to 88%), followed by Mg (varying from 8 up to 19%) and potassium (varying from 2.8% to 4.5%). Plant available levels of these cations ranged from 102 up to 461 mg Mg kg^{-1} and from 81 up to 180 mg K kg^{-1}. In correspondence to the variation in organic matter, the total N content increased from 1542 on sandy maize soils to 6106 mg N kg^{-1} in peaty grassland soils. This resulted in median C-to-N ratios varying between 10.2 up to 14.2. Plant available phosphor was quite high with median concentrations ranging between 5.6 and 5.9 mg P kg^{-1}, using

median values per soil type and land use. The median ammonium lactate extractable P varied from 49 up to 65 mg P_2O_5 100g^{-1}. Similarly, the median water extractable P varied from 52 up to 67 mg P_2O_5 L^{-1}. The potential mineralizable N pool was remarkably constant across soil type and land use groupings, with median values ranging from 218 up to 245 mg N kg^{-1}.

Variations

The magnitude of variation, which was quantified as quartile coefficient of dispersion (QCD), was large for variables such as clay content, silt content, $CaCO_3$, and CEC, and small for pH and availability of N, P, K (Table S4). Eta-square values of ANOVA test were much larger for soil type than land use for most soil properties, showing that among-soil-type variation is larger than among-land-use variation (Table S4, Figure S 3). This indicates that Dutch agricultural soils are on average fertile due to high (historical) nutrient inputs, and that soil type (reflecting soil origin and formation) is the main driver for the variation in soil properties.

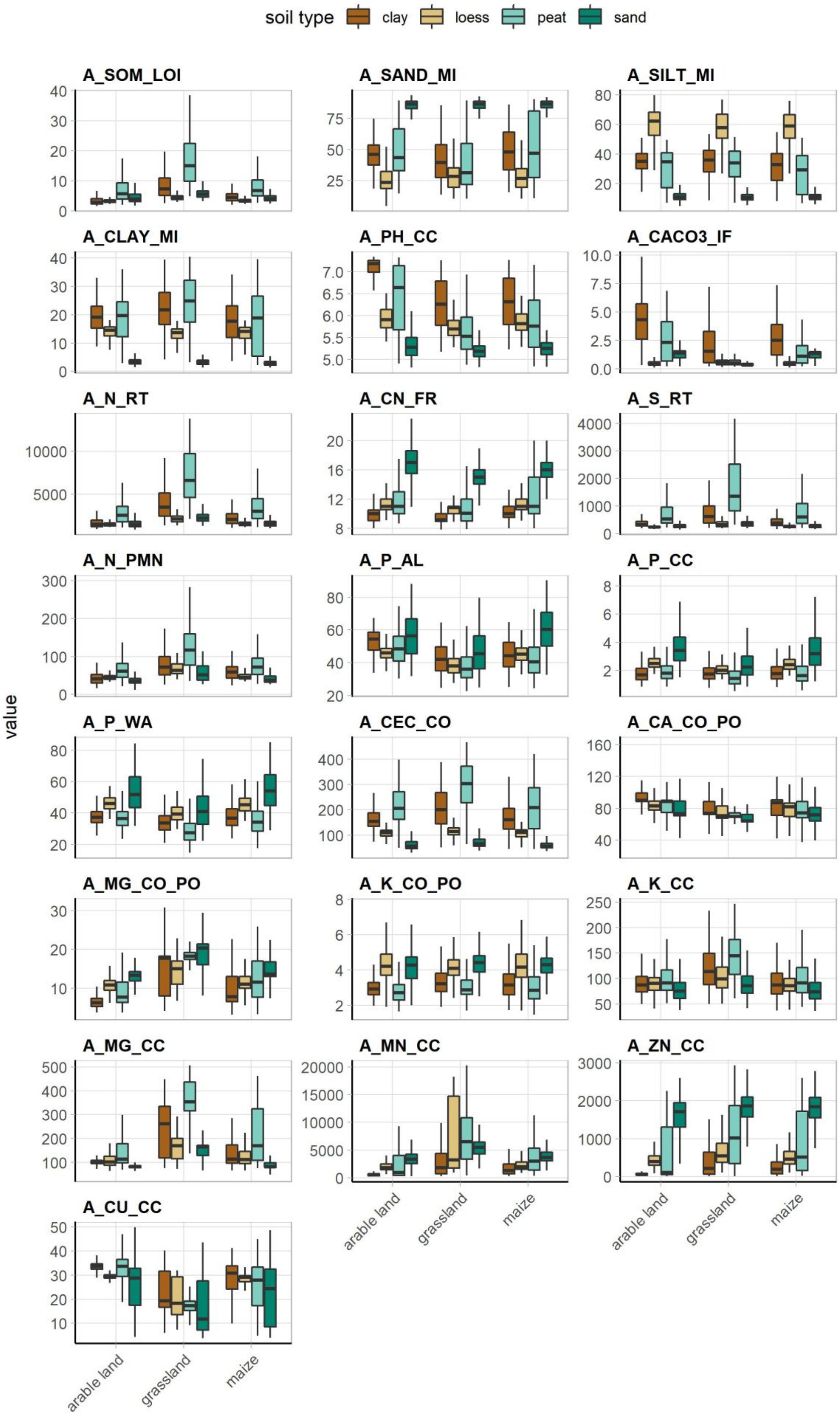

Figure S 3. Median and quantiles of soil properties, separately shown for different soil types and land use. N=762.518 fields. See Table S2 for description and units of each soil property

Table S4. Magnitude of among-field variation of soil properties derived from laboratory analysis, and effects of soil and land use on the variation in 762518 agricultural fields in the Netherlands. Magnitude of variation is quantified with quartile coefficient of dispersion (QCD). Effects of soil type (sand/loess/clay/peat) and land use (grassland/arable land/maize) were quantified with eta-square values of ANOVA test.

variable	description	unit	QCD	eta^2 soil	eta^2 land use
A_SOM_LOI	Organic matter	%	0.93	0.44	0.15
A_SAND_MI	Sand content	%	0.71	0.67	0.00
A_SILT_MI	Silt content	%	1.61	0.65	0.00
A_CLAY_MI	Clay content	%	2.52	0.68	0.01
A_PH_CC	Soil acidity	-	0.18	0.57	0.08
A_N_RT	Total N content	mg N/kg	1.04	0.50	0.15
A_CN_FR	Soil C:N ratio	ratio	0.45	0.66	0.02
A_S_RT	S soil reserves	mg S/kg	1.31	0.54	0.06
A_N_PMN	Microbial activity	mg N/kg	0.90	0.34	0.13
A_P_AL	P soil reserves (ammonium lactate extractable)	mg P_2O_5/100 g	0.46	0.15	0.13
A_P_CC	P plant available	mg P/kg	0.72	0.26	0.05
A_P_WA	P water (water extractable)	mg P_2O_5/ L	0.47	0.32	0.09
A_CEC_CO	Cation exchange capacity	mmol+/kg	1.51	0.65	0.02
A_CA_CO_PO	Ca occupancy CEC	%	0.31	0.10	0.08
A_MG_CO_PO	Mg occupancy CEC	%	0.50	0.19	0.22
A_K_CO_PO	K occupancy CEC	%	0.45	0.39	0.01
A_K_CC	K plant available	mg K/kg	0.59	0.28	0.05
A_MG_CC	Mg plant available	mg Mg/kg	1.27	0.48	0.14
A_MN_CC	Mn plant available	ug Mn/kg	1.08	0.37	0.12
A_ZN_CC	Zn plant available	ug Zn/kg	1.24	0.52	0.02
A_CU_CC	Cu plant available	ug Cu/kg	1.07	0.11	0.14

D. Supplementary figures and tables of study of Dutch agricultural fields

Patterns in soil indicator values

Bottlenecks of soil indicators of 21 soil functions are summarized in Table S5.

Table S5. Bottlenecks in soil indicators of Dutch agricultural fields. Two metrics to reflect the performance of each soil indicator are shown; % fields poor: percentage of fields (out of total 762.518 fields) which scored poor (<0.5), % field bottlenecked: percentage of fields for which the indicator scored the worst among all indicators within the category (chemical physical, biological or environmental). When the worst score of a field is higher than 0.7, the field was excluded from the calculation of % field bottlenecked. % field bottlenecked is also calculated per land use and soil type (C: clay, L: loess, P: peat, S: sand) separately.

Soil indicator	Description	% fields poor	% field bottlenecked	% field bottlenecked, per soil type and land use											
				------Arable land------				------Grassland------				--------Maize--------			
				C	L	P	S	C	L	P	S	C	L	P	S
Chemical soil functions															
I_C_CEC	Cation Exchange Capacity	10.3	5.9	0	0	0	42	0	0	0	2	2	1	1	23
I_C_CU	Copper availability	2.6	3.2	1	0	1	0	8	0	8	0	2	1	4	0
I_C_K	Potassium availability	7.6	6.6	0	4	1	4	3	1	2	1	17	14	21	44
I_C_MG	Magnesium availability	2.5	1.6	0	0	0	1	3	0	4	0	0	0	0	1
I_C_N	Nitrogen availability	1.6	1	0	0	0	9	0	0	0	0	0	0	1	3
I_C_P	Phosphorus availability	11.2	12.1	0	0	4	1	11	0	38	5	2	1	14	1
I_C_PH	Soil acidity	4.2	2.9	1	40	2	12	0	0	1	3	1	7	3	5
I_C_S	Sulphur availability	49	47.6	40	39	72	21	50	93	20	85	22	38	19	17
I_C_ZN	Zinc availability	21.3	14.5	57	16	12	9	18	5	14	2	53	38	34	4
Physical soil functions															
I_P_CEC	Aggregate stability	3.3	1.2	2	1	2	3	1	1	0	1	5	2	3	3
I_P_CO	Compaction	49.7	28.6	85	89	57	37	22	27	7	17	73	85	47	44
I_P_CR	Crumbability	0	0	0	0	0	0	0	0	0	0	0	0	0	0
I_P_DS	Droughtstress	4.9	2.2	5	1	6	8	1	1	1	1	3	2	3	3
I_P_DU	Wind erosion	24.3	13.8	1	0	9	44	1	0	1	17	5	0	14	47
I_P_SE	Soil sealing	0.2	0.1	1	1	0	0	0	0	0	0	1	1	0	0
I_P_WRI	Water retention	0.1	0.1	0	0	1	0	0	0	0	0	0	0	0	0
I_P_WS	Wetnessstress	20.9	4.5	3	0	20	8	3	0	7	2	10	0	30	4
Biological soil functions															
I_B_DI	Disease / pest resistance	6.6	10.3	41	55	17	22	6	8	0	2	22	43	6	20
I_B_SF	Soil life activity	4.3	3.6	23	0	1	12	1	0	0	1	2	0	0	1
Environmental soil functions															

I_E_NGW	Nitrogen retention for ground water	16.8	22.5	9	61	7	5	6	95	1	46	31	96	35	35
I_E_NSW	Nitrogen retention for surface water	9.8	7.3	4	0	14	4	24	0	1	2	19	1	19	14

OSI sub-scores of Dutch agricultural fields

OSI sub-scores of four categories for all agricultural fields in the Netherlands are shown in Figure S 4.

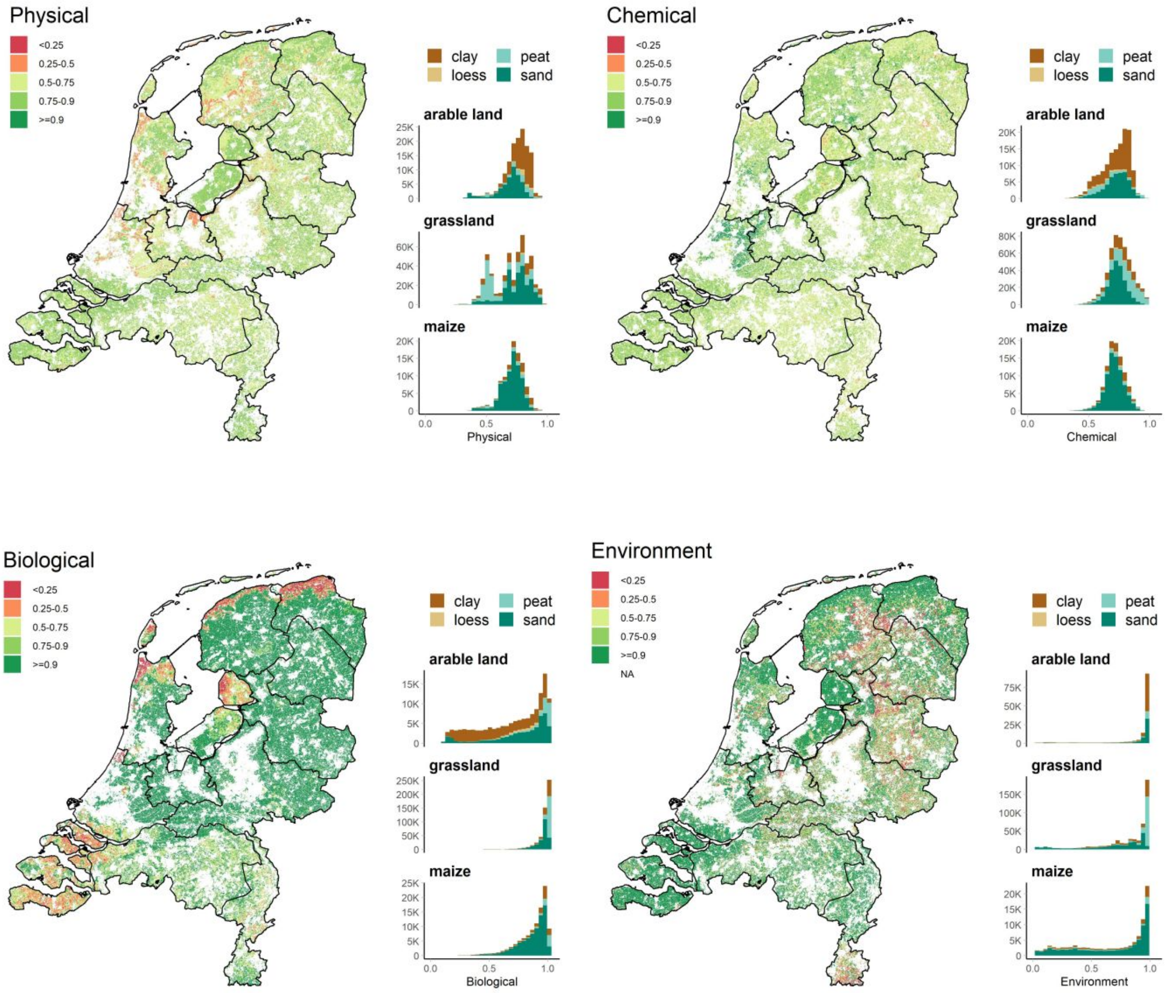

Figure S 4. Spatial distribution of the OSI sub-scores of composing category: physical, chemical, biological and environmental sub-scores. Histograms are shown separately for different land use and for soil type.

E. Analysis on indicator complementarity and redundancy

Methods

Using the dataset of all Dutch agricultural fields (N = 762518), relationship among soil indicators were tested with Spearman's correlation test. Additionally, in order to extract the major axes of variation and therewith understand the relations between the multiple indicators, principal component analysis (PCA) was conducted based on the correlation matrix of indicator values of 21 soil functions (i.e. excluding the soil function management).

Results

Rank correlation coefficients were computed for every pair of 21 soil indicators of chemical, physical, biological and environmental category. Correlation among 21 soil indicators was weak to moderate (Figure S 5 left), with only 9 pairs (out of total 210 pairs) having a positive correlation coefficient (ρ) higher than 0.5 and 6 pairs having a negative ρ correlation coefficient lower than -0.5. This indicates that the soil indicators of the OSI are complementary rather dan redundant. Strong positive correlation was observed between the two environmental indicators (I_E_NGW and I_E_NSW, ρ = 0.84), between two biological indicators (I_B_SF and I_B_DI, ρ = 0.72), between I_C_N and I_B_DI (ρ = 0.77), and between I_C_N and I_P_SE (ρ = 0.72). All of these indicators are calculated from (and therefore strongly related to) soil C or N content as input. Strong negative correlation was observed between I_P_SE and I_P_DU (ρ = - 0.85), both are largely regulated by clay content.

PCA analysis was conducted to examine the relations of the 21 soil indicators on a multidimensional space. The first 8 axes of PCA accounted for 70% of the total variation, and there was no dominant component which explained a large portion of the variation: the first and second axis accounted for 18% and 13% of total variation, respectively. The loadings of the soil indicators spread over the biplot of axis 1 and axis 2 without much overlap (Figure S 5 right), indicating minimal redundancy among the indicators even on the multidimensional space. Environmental indicators (I_E_NSW and I_E_NGW) locate on the opposite sides of the indicators reflecting high SOM and N content (e.g. I_C_N, I_B_DI, I_P_SE), but they are not completely isolated from other soil indicators on the biplot; for example, they locate nearby the indicators for wind erosion and aggregate stability. This indicates that improving soil quality for sustainable crop production is not necessarily at the expense of environment, but can make a positive contribution to reducing environmental impacts.

The grassland fields tend to distribute on the right-hand side of the PCA biplot, whereas maize and arable fields tend to distribute on the left-hand side (Figure S 6). Clay soils tend to distribute on the bottom of the biplot, where is associated with relatively high indicator values of CEC, S, and N retention to groundwater. Although the patterns associated with land use and soil type were evident, each group covers a wide range on the multidimensional indicator space, indicating that the OSI is able to separate gradients of soil quality within each group.

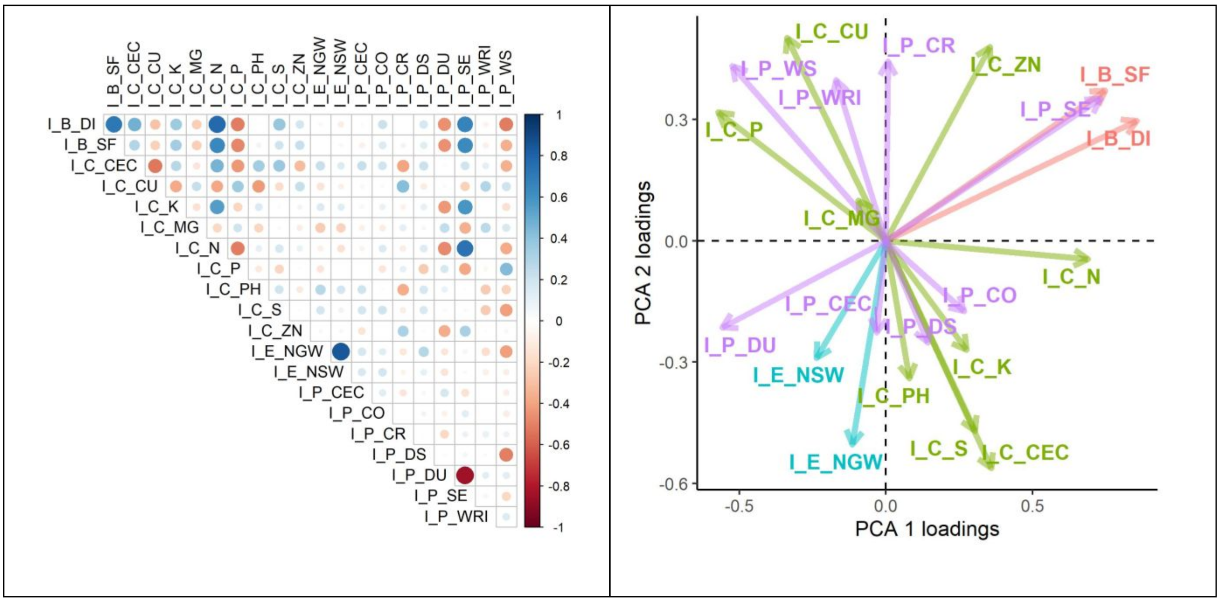

Figure S 5. Spearman's correlation (left) between indicators values of 21 soil functions and PCA-biplot of loadings (right) among them. SeeTable S for abbreviation of the soil indicators

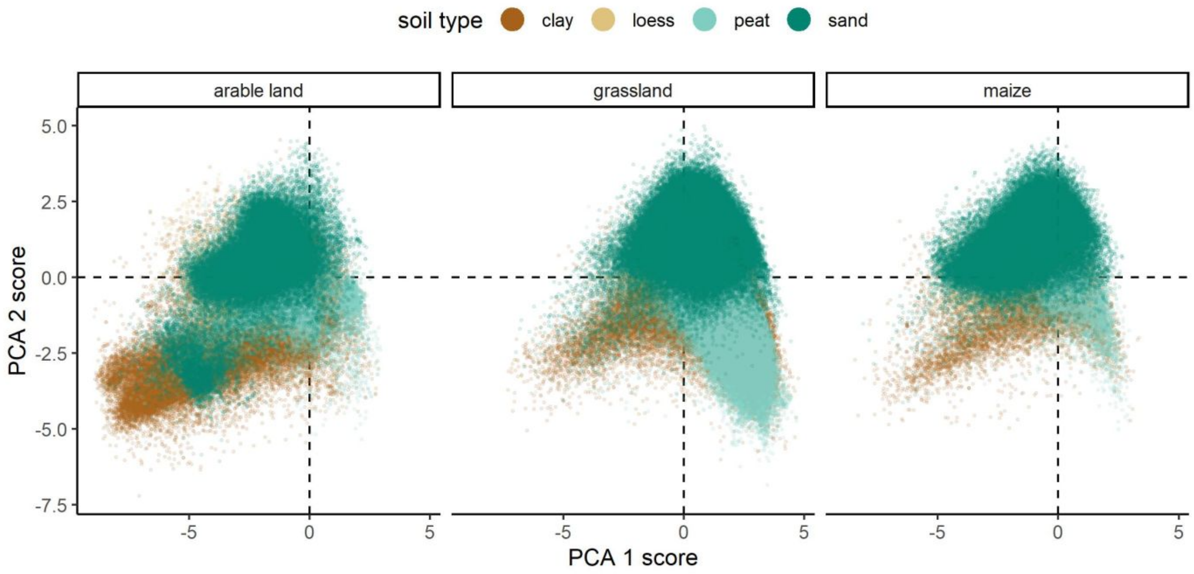

Figure S 6. Scores of 762518 fields on PCA biplot of axis 1 and 2.

F. Error propagation analysis

Methods

An error propagation study was conducted to test sensitivity of the final OSI score against changes in soil property values, as well as against aggregation methods.

The OSI scores and indicators were first computed based on median values of all soil properties (as reference), and then computed using the same input dataset with only one soil property replaced with an extreme value (95th and 5th percentile of all Dutch fields). The soil properties examined are 22 numerical parameters measured in laboratory plus three categorical parameters (groundwater level, compaction risk, soil type for water stress evaluation). For the categorical parameters, the classes used as 'extreme' and median conditions were as follows: groundwater level 'I' for high, "IV" for median, and 'VIII' for low condition; compaction risk 'very large' for high, 'intermediate' for median, and 'very limited' for low condition; soil type "ABl" for high, "HD21" for median, and "FKk|B" for low condition. The deviation in indicator values and OSI scores due to the replacement of a single soil property was quantified, for three land use types and four soil types separately. In addition, to test how the aggregation methods affect the error propagation, the same analysis was conducted with a dataset in which the OSI sub-scores and scores were calculated without weighing factors but as arithmetic means of all soil indicators.

Results

To assess the sensitivity of the OSI, response of OSI scores to a change in single input value was examined. The final score was not drastically influenced by an extreme value of a soil property (Figure S 7 left). A relatively large deviation in the final score was observed when SOM content was very high, especially in arable fields (change in the score up to -0.18). This high sensitivity to SOM was expected, as SOM is the input parameter for many soil indicators (N, K, Mg, S, sealing, disease resistance, N retention to groundwater and surface water). Accordingly, SOM affect both chemical, physical, biological, and environmental sub-scores (Figure S 7 right). Other soil properties which substantially influence the OSI final score were groundwater level, soil N content, soil S content, C:N ratio, and CEC. Errors or changes in these soil properties have relatively large influence on the final OSI score.

Two soil properties are currently retrieved from a coarse national map (B_SC_WENR and B_HELP_WENR) and therefore they are prone to an error. The effect of B_SC_WENR on the soil function compaction is large (-0.4 to +0.4 change), yet its influence on physical sub-score (up to - 0.18) and final score (up to - 0.05) was limited. The effect of B_HELP_WENR on indicators and scores was even smaller.

In the current OSI, the soil indicators are aggregated into sub-scores using weighing factors, which give relatively higher weight for lower scores. When the aggregation was done with simple arithmetic mean of all indicators (i.e. without weighing factors), the changes caused by an extreme value of single soil properties became smaller: the final score deviated only between -0.15 and +0.12 (Figure S 8). In particular, the change in the chemical sub-score was hardly visible, varying only between -0.15 to +0.11. The changes in the chemical sub-score were much larger when the default aggregation methods were used (which ranges between -0.39 and +0.37), indicating that the default aggregation methods can better capture the signal of deviating soil function in the final scores. This illustrates a large influence of aggregation methods on the OSI scores and sub-scores and supports the relevance of introducing the weighing factor in the aggregation step.

It should also be noted that, when the values of all soil properties are the same, the OSI final score differs considerably among soil types and land use, especially for the environmental sub-scores (Figure S 9). This comes from the use of different formulas and/or parameter values among soil types or land use for many indicators (Table S3 in Supporting Information A). This is necessary because, by setting landuse- and soil-specific optimum values, it is in principle possible for any field to reach the desired optimum score. Proper assignment of land use and soil type is thus important to calculate a relevant OSI final score for a specific setting.

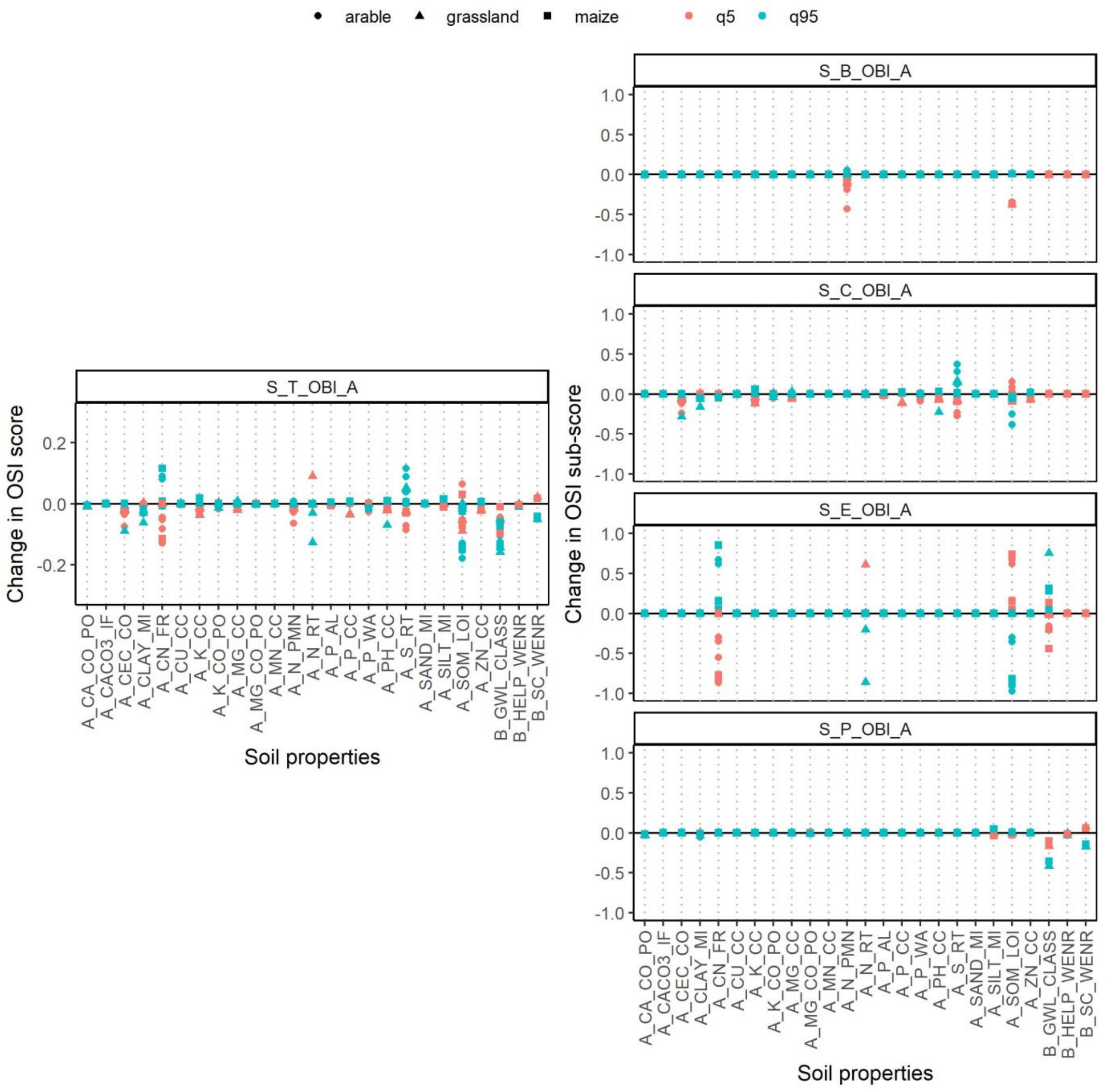

Figure S 7. *Change in the OSI final score (left) and sub-scores (right) when the value of a soil property is replaced with an extreme value, in comparison to when the OSI score is calculated with median values of all soil properties. The extremely low and high values were the 5th quantile (q5) and 95th quantile (q95) values of all Dutch agricultural fields. Positive change means that the OSI score becomes higher due to the replacement of the single soil property value. The OSI score was computed for 3 land use (shown with different symbols) and 4 soil types separately. Sub-scores are: biological (S_B_OBI_A), chemical (S_C_OBI_A), environmental (S_E_OBI_A), and physical (S_P_OBI_A) scores.*

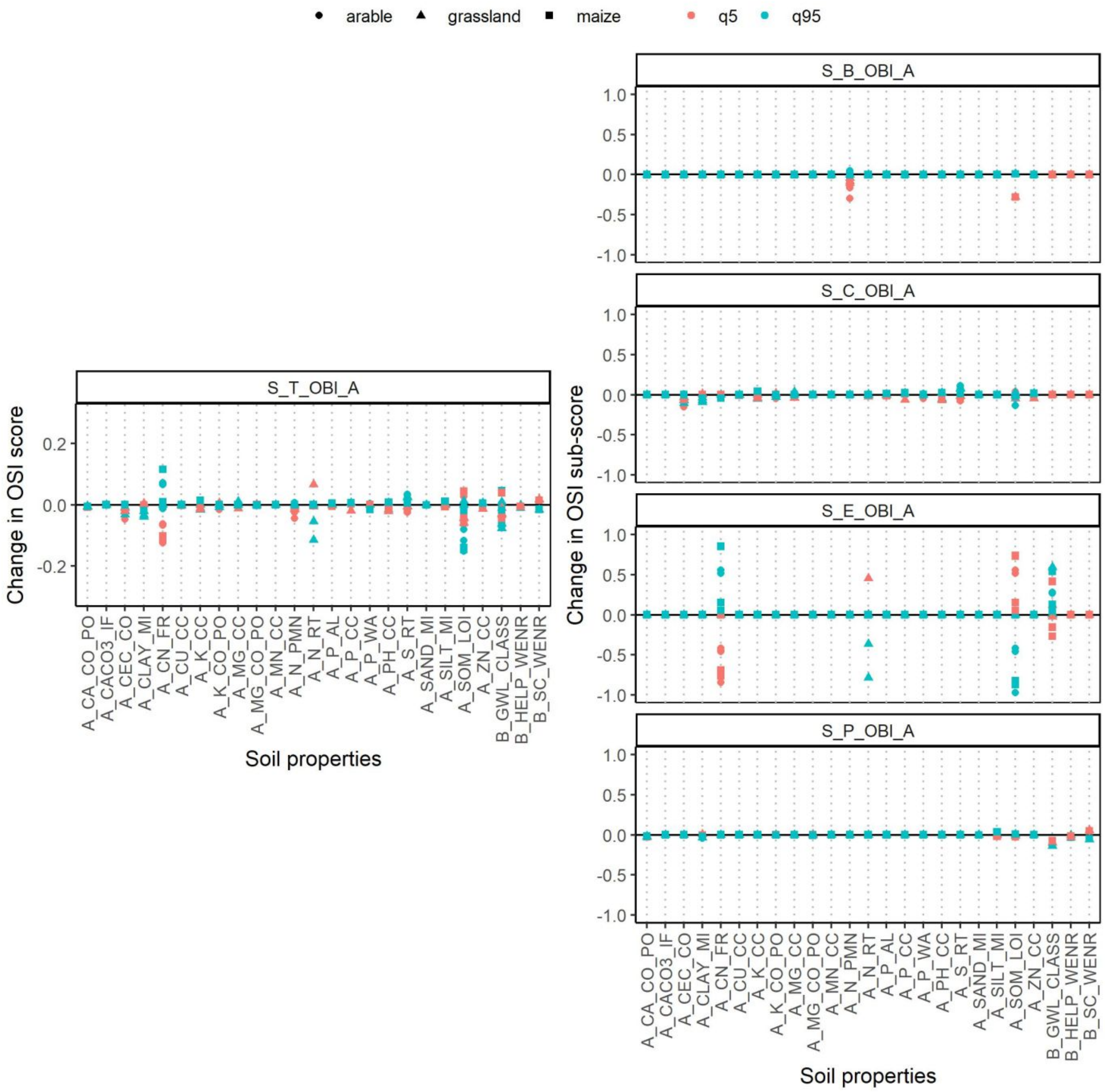

Figure S 8. Same as Figure S 7., except the aggregation method to calculate sub-scores from soil indicators. Here, the sub-scores were computed as arithmetic mean of all soil indicators within the category, instead of using the weighing factors.

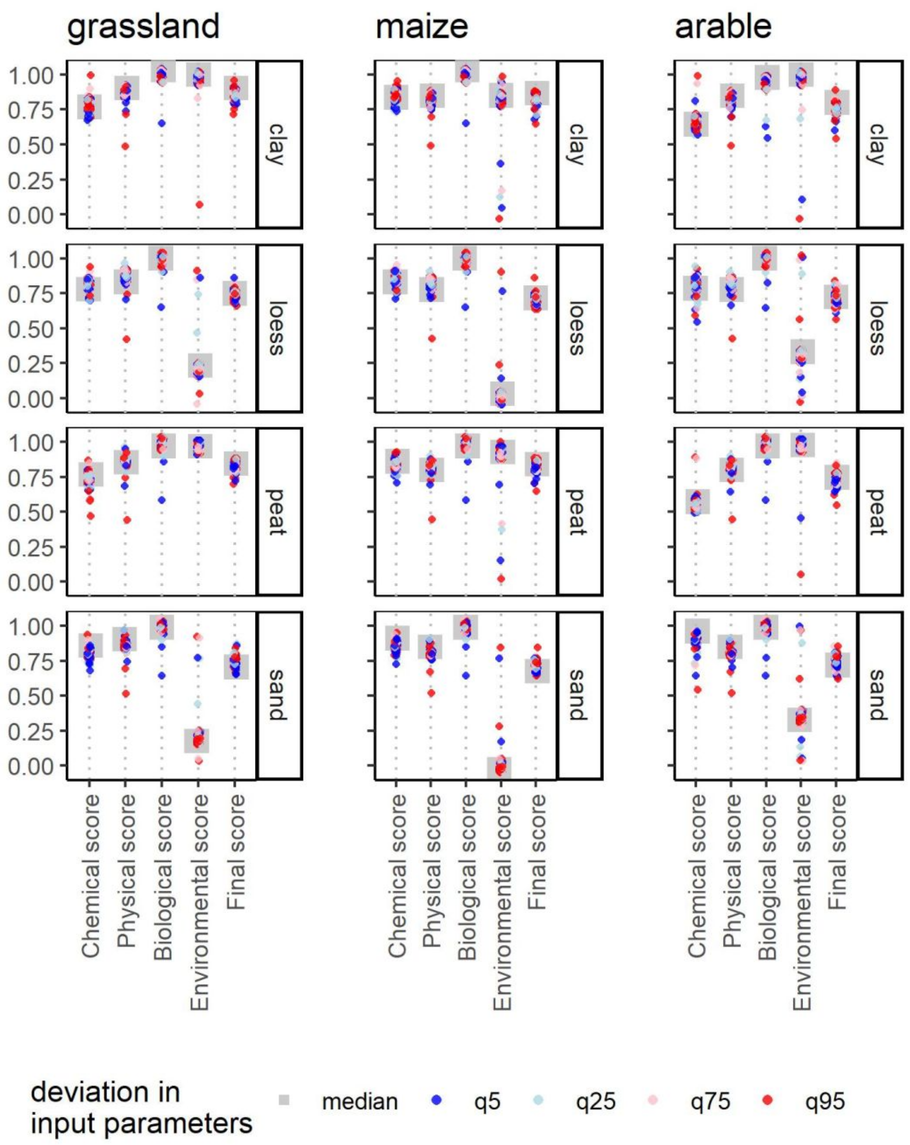

Figure S 9. Influence of deviation in a single input parameter (soil properties) on OSI scores and sub-scores. Gray squares show the scores when calculated with median values of Dutch agricultural fields for all soil properties. Circles show the scores when an extreme value (95th or 5th quantile of Dutch agricultural fields) was used for a soil property whereas median values were used for the other soil properties. This was repeated for 26 soil properties (i.e. 23 numerical soil properties, B_HELP_WENR, B_SC_WENR, B_GWL_CLASS).

G. OSI test results for 22 field sites

OSI indicators and scores for 11 pairs of case study fields, as well as scores of VSA (visual soil assessment) are shown in Figure S10.

Case 10: [good] arable land on sand, [poor] arable land on sand

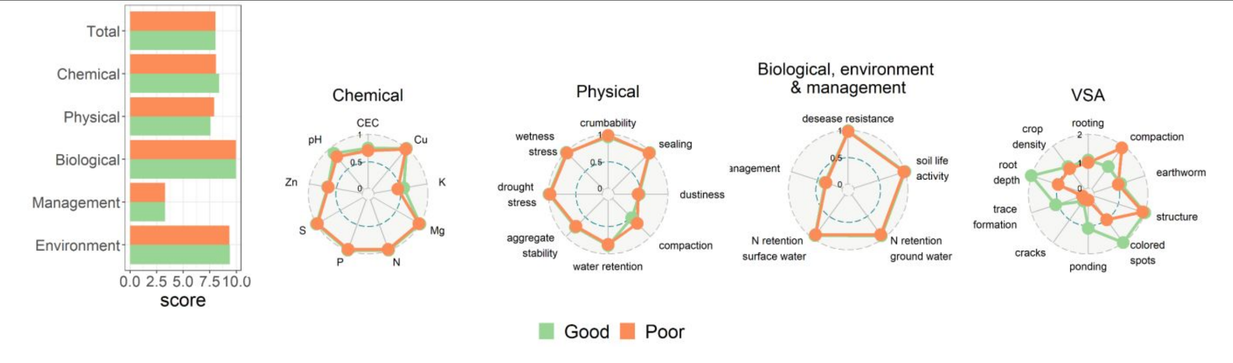

Case 11: [good] mais on sand, [poor] grassland on sand

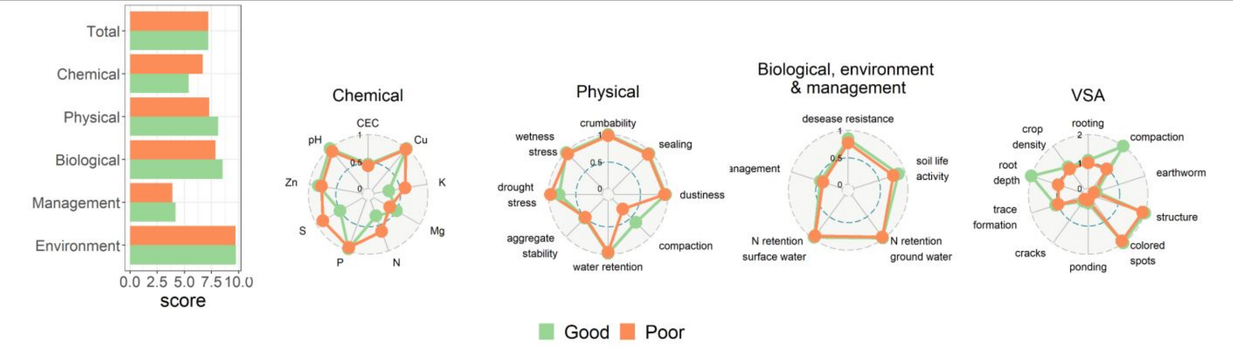

Case 12: [good] arable land on clay, [poor] arable land on clay

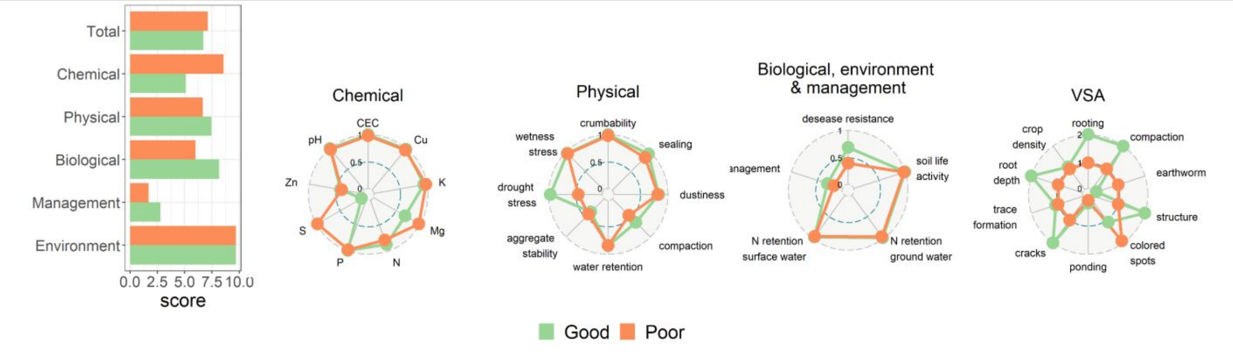

Figure S 10

Case 1: [good] arable land on clay, [poor] arable land on clay

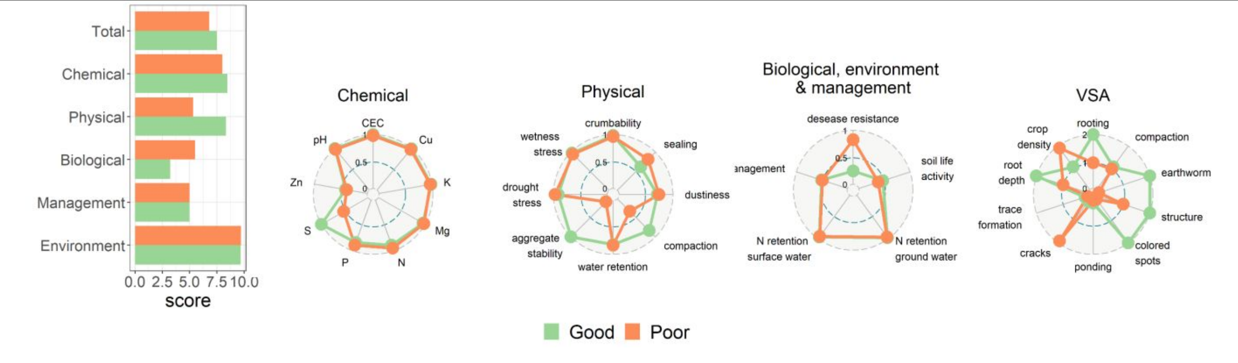

Case 2: [good] arable land on sand, [poor] arable land on sand

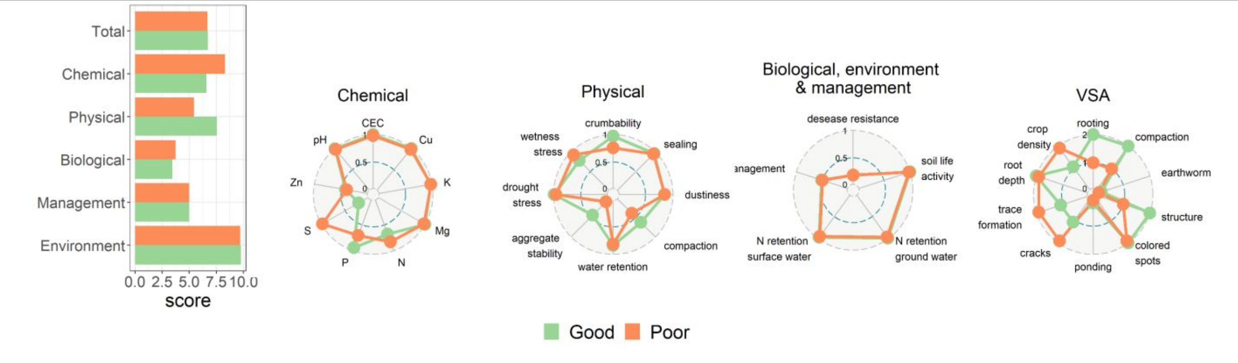

Case 3: [good] arable land on sand, [poor] arable land on sand

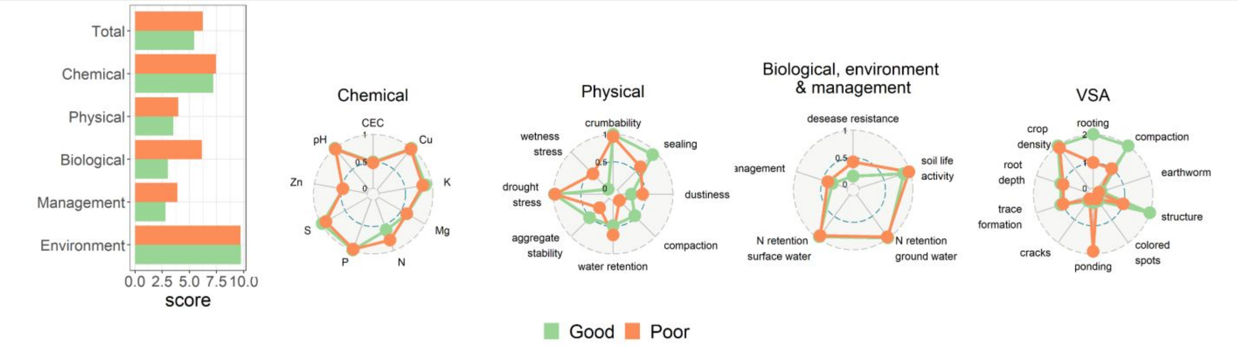

Case 4: [good] grassland on clay, [poor] grassland on clay

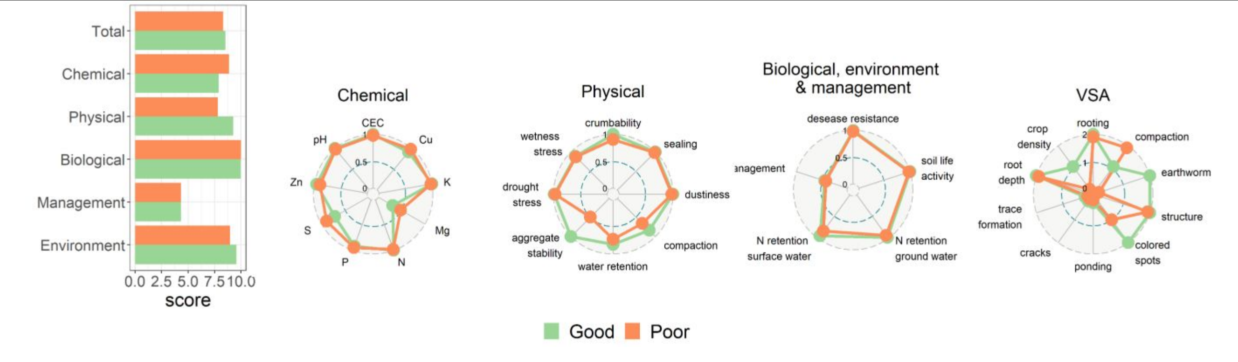

Case 5: [good] arable land on clay, [poor] grassland on clay

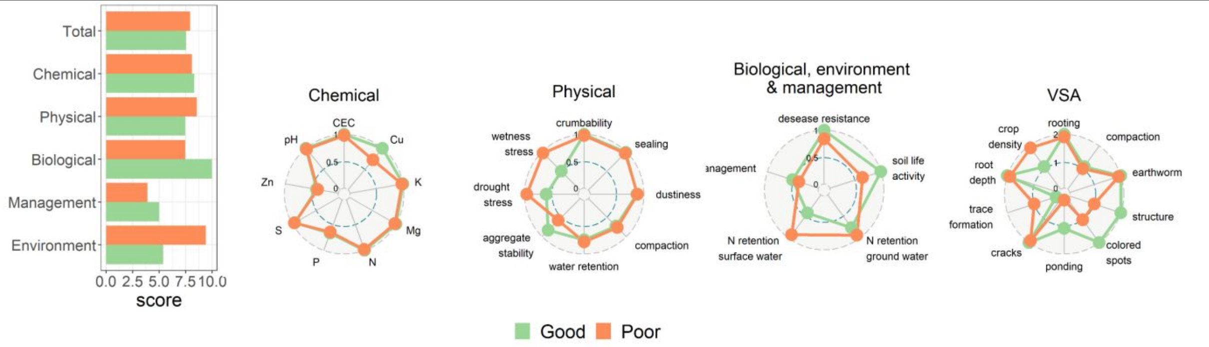

Case 6: [good] arable land on sand, [poor] arable land on sand

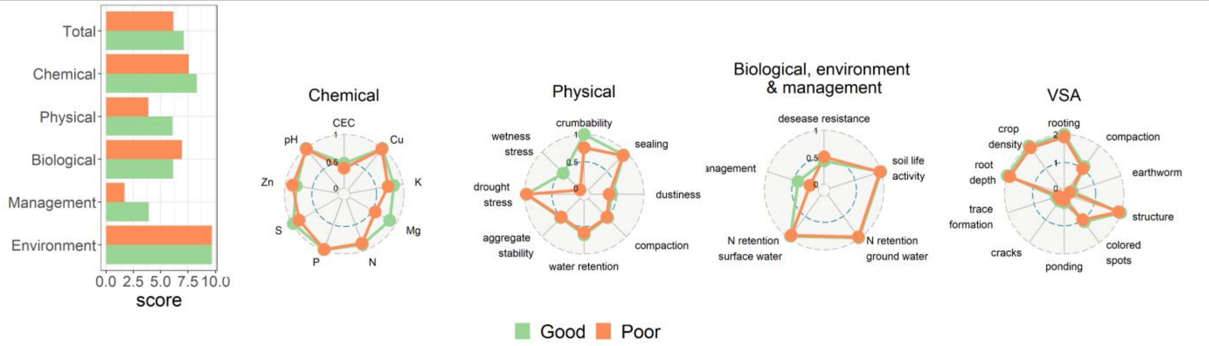

Case 8: [good] grassland on clay, [poor] arable land on sand

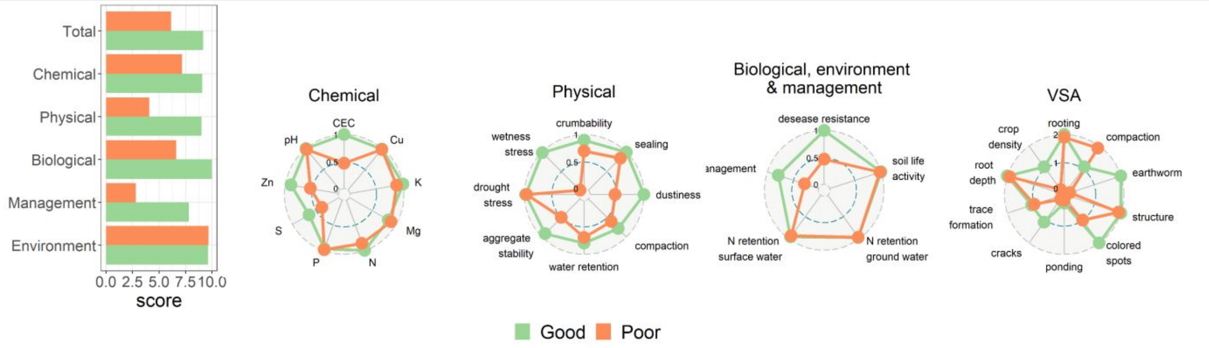

Case 9: [Good] grassland on clay, [poor] grassland on clay

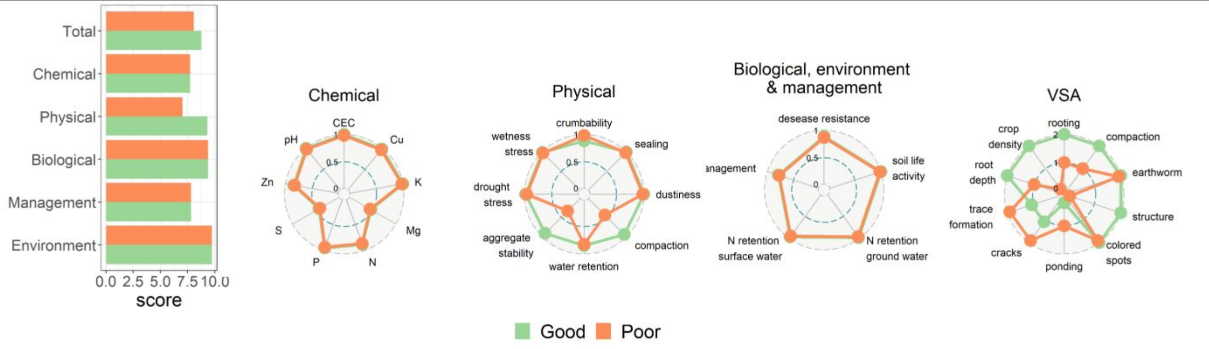

Case 10: [good] arable land on sand, [poor] arable land on sand

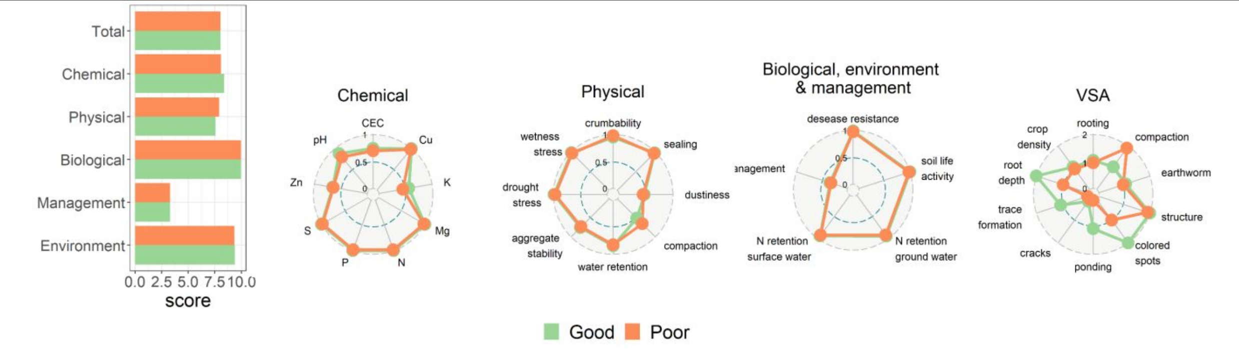

Case 11: [good] mais on sand, [poor] grassland on sand

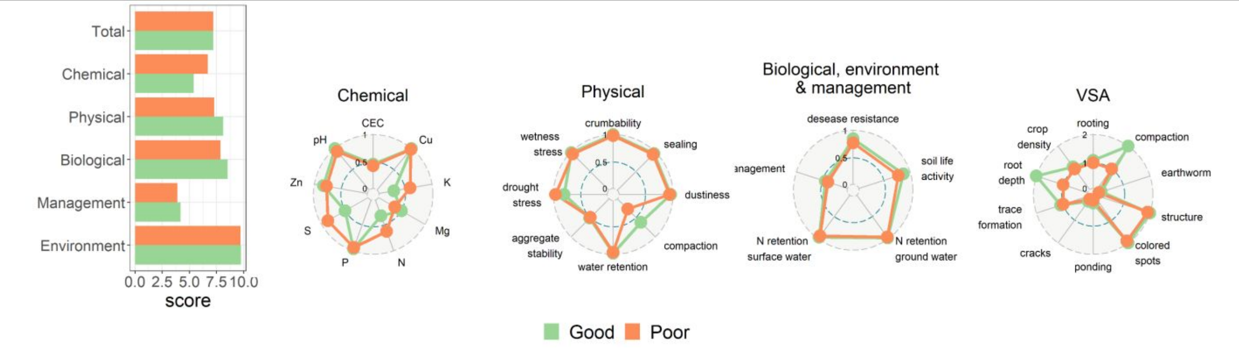

Case 12: [good] arable land on clay, [poor] arable land on clay

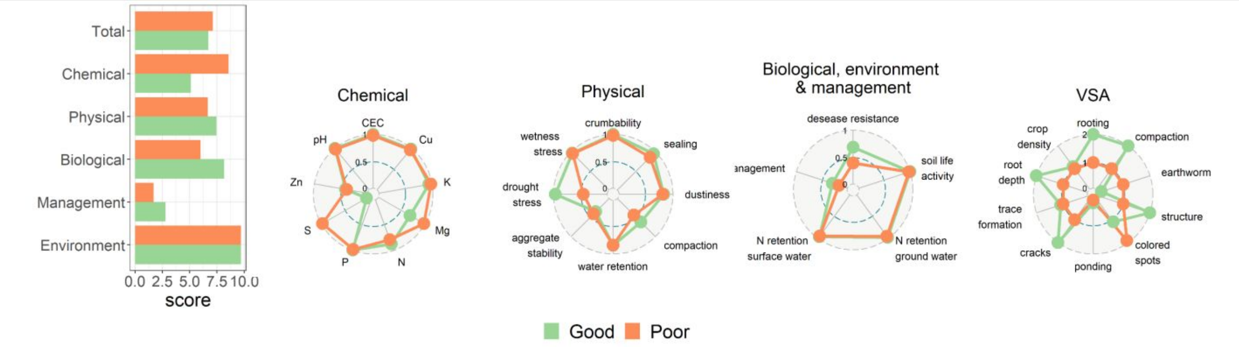

Figure S 10. OSI test results of 11 pairs of case study fields. Bar graph shows Final OSI score ('Total') as well as sub scores of five categories. Spider graphs show indicator values of 22 soil functions plus scores of visual soil assessment (VSA).

H. Comparison with other soil assessment frameworks

A number of soil assessment frameworks exist, each with its own advantages and disadvantages. Extensive comparisons were made elsewhere (Bünemann et al., 2018; Rinot et al., 2019). Here we focus on the difference between the OSI and the other framework and identify pros and cons of the OSI.

The attributes of existing soil assessment frameworks applicable at the field level are compared in Table S6. The comparison was made in terms of: included soil functions, necessary input parameters to evaluate the soil functions, method of evaluation, spatial scale, objective of the framework, whether the framework provides advice for management measures, and aggregation method to a final score.

The covered soil functions vary among frameworks, and the choice depends on the objective for which the soil quality is assessed for. Some frameworks offer options to choose the objective of the assessment, and therewith adjust the soil functions to be examined (e.g. SMAF). The OSI defined a set of soil functions which are related to sustainable crop production, but thanks to the modular system developed in open-source environment, users can add or delete specific soil functions.

The required input parameters are 25 (of which 19 are soil parameters) for the current Dutch implementation of the OSI, which is more than the average number of indicators (11) of various frameworks reviewed by Bunemann et al (2018). A notable feature of the OSI is that it uses routine laboratory data of soil analysis, which is evaluated regularly for all agricultural fields in the Netherlands. This makes the applicability of the OSI to the farmers high, regardless of the relatively large number of input parameters.

The evaluation system (i.e. the way to translate original parameter values to assessment metrics) varies among frameworks as well. The common approaches are expert judgement system and relative position in relation to comparable or reference fields. The latter method is inevitably tied to the challenge of proving that the reference fields represent the “good” condition. The former approach, expert judgement, is more widely used. The OSI also uses a sort of expert judgement, but it primarily uses the knowledge from various (long-term) fertilization experiments on which national fertilization guideline is based. The knowledge is used to define scoring curve and quantify the ‘distance to target’ in the standardized scale between 0 and 1.

Not all frameworks offer a single, final score to evaluate the soil quality. The OSI offers the final score, with the scope that such final evaluation is useful for third parties (e.g. financial sector, governments) to valorise soil quality. To compute the final score, aggregation of scores of different soil functions is necessary. Aggregation methods varies from simple unweighted sum to weighted sum. The OSI uses 3-step aggregation approaches with different weighing factors (see Supporting Information B), and aggregation approach can be adjusted according to the scope of the application. The aggregation method influences the final score to a large extent (Supporting Information F), and our current choice of the aggregation methods in the OSI is effective to capture the signal of poorly-performing soil function in the final score.

Finally, a few frameworks have a direct link to farming measures. SoilNavigator is especially designed to offer management advice and evaluate the effects. The OSI also offers advice to improve soil quality, for three different categories of soil functions (chemical/physical/biological) separately. Since

any global or national target in sustainable agriculture calls for local actions taken on field levels, the ability of a soil assessment tool to bridge between global targets and local actions is a prerequisite.

To summarize, the OSI attempts to use the best building blocks of existing frameworks, with an emphasize on its scalability (i.e. leveraging available agronomic data and knowledge) and operationality (i.e. open-source modular architecture to allow users to make the framework applicable for their own region, coupling with advice for farming practices). We claim that the OSI offers a framework which is a step forward from the existing ones because 1) it provides a generic, robust frame built on proven concepts and elements from the past studies, 2) it is successfully implemented for Dutch agricultural fields on local and national level at minimum costs, and 3) it has a serious perspective on valorisation, as evidenced by the collaboration with financial and governmental parties and farmers.

Table S6. Comparison of soil assessment frameworks.

Tool	Reference	Covered sol functions	Source of Input parameters	Evaluation method	Spatial scale	Objective	Provide advice for measures	Aggregation to final score	Remarks
Open Soil Index (OSI)	This study	Chemical (9), physical (8), Biological (2), environmental (2), management (1) indicators	Routine soil measurements, satellite data, national database	0-1, quantitative scoring function (Distance to target) based on agronomical experiments	Fields to national	Assessment of soil health in terms of sustainable crop production	yes	3-step weighted, non-linear aggregation	Open source<br><br>Modular system
SoilNavigator	(Debeljak et al., 2019)	Primary productivity / water purification / climate regulation / soil biodiversity / nutrient provision	Soil measurements, climatic and geographic data, management practices	Qualitative (low / medium / high), based on expert judgement	Field	Decision support for assessment and management of soil functions	yes	NA	Interactive GUI available<br><br>Developed within EU Horizon 2020 project LANDMARK (Land Management Assessment Research Knowledge base)
Comprehensive Assessment of Soil Health (CASH)	(Moebius-Clune et al., 2016)	Chemical (7), physical (4), biological (5) indicators	Soil measurements	0-1, Scoring function based on existing soil fertility recommendation systems (for pH and nutrients), or on dataset cumulative normal distribution (for the rest)	Field to regional	Assessment of soil health	no	Unweighted sum of all scores	
Soil management assessment framework (SMAF)	(Andrews et al., 2004; Wienhold et al., 2009)	Chemical, physical, biological indicators (4 – 8 indicators, depends on user-defined goal)	Minimum dataset (MDS), selected from 80+ potential soil parameters	0-1, scoring function based on past studies	Field	Assessment of soil quality for different management goals (Maximize productivity / waste recycling /	no	Unweighted sum of all scores	

						environmental protection)			
Bobi	(Rutgers et al., 2012; van Wijnen et al., 2012)	User defined and site-specific	Soil measurements	Relative to reference (i.e. maximum ecological potential) condition	Field to national	Quantification of ecosystem service	no	Exponent of sum of logs	
iSQAPER	https://isqaper-is.eu/	Chemical (6), physical (7), biological (7) indicaors	global soil maps and databases (replaceable with local field data)	Relative score within pedo-climatic zone	Field	Assessment of soil quality and threat, provision of management advice	yes	NA	The interactive app 'SQAPP' is available<br><br>Developed within EU Horizon2020 project iSQAPER (Interactive Soil Quality Assessment in Europe and China for Agricultural Productivity and Environmental Resilience)
Biofunctool	(Thoumazeau et al., 2019b, 2019a)	Structure maintenance, nutrient cycling, carbon transformation	12 in-field soil measurements	0-1, scoring function	Field	Assessment of soil quality	no	Weighted sum (with weights derived from PCA)	

I. Application of other assessment frameworks

The soil assessment framework CASH (Moebius-Clune et al., 2016) was applied to the 11 pairs of fields used in our second case study.

CASH includes 4 physical, 5 biological, and 7 chemical indicators, plus several extra add-on indicators (Table S7). Most of the CASH indicators require lab measurements of soils. Of the 16 standard indicators of CASH, input parameters were available from Dutch fields (from routine soil laboratory data, open-source national data) for 9 indicators only, mainly chemical indicators. This highlights the limited application possibilities of the CASH on a large scale as it requires many lab measurements of the soils.

Here, for a comparison purpose, we calculated 8 CASH indicators for which input data is available for the Dutch fields and the OSI has comparable indicators. Those are: soil pH, total N, extractable P, extractable K, extractable Mg, extractable Zn, available water capacity (which is called 'water retention' in the OSI), and potentially mineralizable nitrogen ('soil life activity' in the OSI).

The results of CASH are shown along with the corresponding OSI indicator scores (Figure S11). pH and N availability are for some cases more strictly evaluated with CASH. The former is attributed to relatively low threshold values of CASH for pH. N availability was evaluated based on different parameters (total N in CASH and N supply capacity in OSI) and therefore direct comparison is not possible. In general, the order of scores between good and poor fields are identical for the two frameworks: 7 out of 11 pairs have a higher (or identical) average score for good than poor fields with OSI, whereas 6 out of 11 pairs have a higher score for good fields with CASH. Due to the limited size of the dataset and lack of quantitative information to validate the scores, it is difficult to further assess which framework better represents the field condition.

Table S7. Soil indicators included in the CASH framework, as standard package or as add-on options. The method to obtain the input parameters are shown. "Prediction" means that the parameters are predicted using an empirical model from a suite of measured sample parameters.

**Category**	**CASH indicators**	**Method**	**Input data available for Dutch dataset**	**Comparable OSI indicator**
Physical	Wet aggregate stability	Lab measurement (with rain simulator)	N	Aggregate stability
	Available water capacity	Lab measurement (ceramic plates in pressure chamber)/ prediction	Y	Water retention
	Soil Strength 0-15 cm	Field measurement (penetrometer)	N	(Soil compaction)
	Soil Strength 15-45 cm	Field measurement (penetrometer)	N	(Soil compaction)
Biological	Organic matter content	Lab measurement (loss on ignition)	Y	-
	Active carbon content	Lab measurement ($KMnO_4$ extraction)	N	-
	ACE soil protein index	Lab measurement (Autoclaved Citrate Extraction) / prediction	N	(Soil life activity)
	Soil respiration	Lab measurement (4-day incubation)	N	(Soil life activity)
	Root rot rating	Lab measurement (visual rating after 4-week treatment)	N	-
	Potentially mineralizable nitrogen**[1]	Lab measurement (7-day anaerobic incubation at 30 °C)	Y	Soil life activity

Chemical	pH	Lab measurement	Y	pH
	Extractable P	Lab measurement (modified Morgan extraction)	Y	P supply
	Extractable K	Lab measurement (modified Morgan extraction)	Y	K supply
	Extractable Mg	Lab measurement (modified Morgan extraction)	Y	Mg supply
	Extractable Fe	Lab measurement (modified Morgan extraction)	Y	-
	Extractable Mn	Lab measurement (modified Morgan extraction)	Y	-
	Extractable Zn	Lab measurement (modified Morgan extraction)	Y	Zn supply
	Soluble salts*	Lab measurement (Electrical conductivity)	Y	-
	Heavy metal screening*	Lab measurement (acid digestion, ICP)	N	-
	Hot water-soluble boron*	Lab measurement	N	-
	Total C*[*2]	Lab measurement (dry combustion, NDIR)	Y	-
	Total N*[*2]	Lab measurement (dry combustion, thermal conductivity detection)	Y	N supply[*3]

* Add-on indicators
*1: replaced with soil protein and respiration in the CASH package, yet still measured and evaluated as add-on indicator
*2: not included in the standard CASH package, although factsheet and evaluation function are available
*3: OSI indicator is evaluated on N supply capacity, not total N

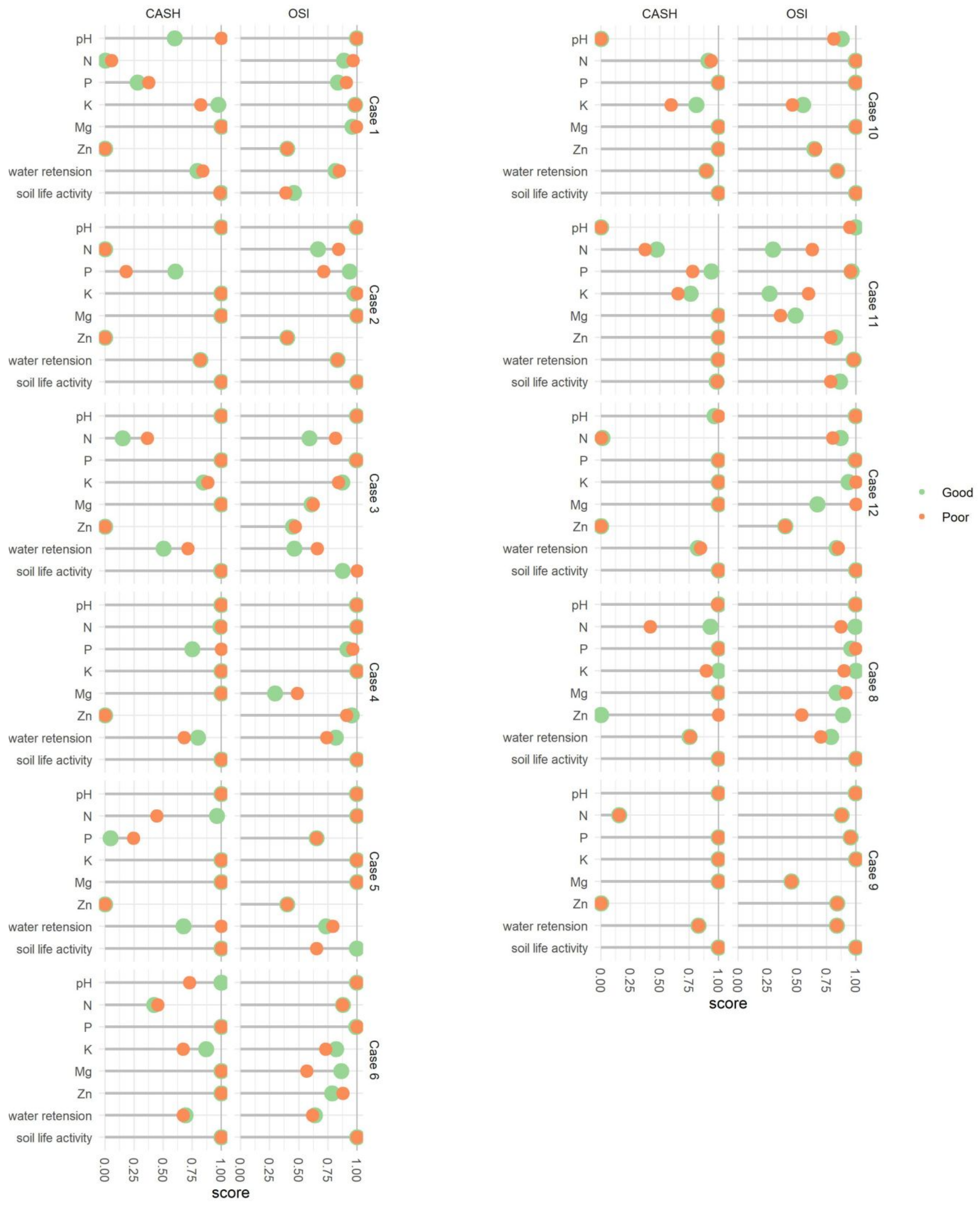

Figure S11. Soil quality index assessed with CASH and OSI for 11 pairs of fields. Full assessment of these fields with OSI was presented in Appendix G.

J. Availability of soil data and agronomic knowledge base worldwide

Wherever agriculture is practiced, agronomic knowledge is available in some form or another. Structured data and knowledge basis becomes increasingly available, led by public initiatives such as FAO but also by private sectors, due to the increased interest in precision agriculture and advanced technologies to collect and process data.

Here we present a brief overview of availability of agricultural soil data and knowledge bases all over the world.

Soil data and laboratories

Most countries have agricultural laboratories to provide advice for farmers. FAO's initiatives within Global Soil Partnership (GSP) plays a pivotal role in coordinating and harmonizing soil laboratories in the world. Next to the nationally mandated institution INSII (International Network of Soil Information Institutions), FAO launched a network of national soil laboratories, GLOSOLAN, to share information and experiences as well as to develop harmonized standards and data among countries. The laboratories spread literally all over the world, as shown in the map below (Figure S12). The GSNmap, another initiative within the GSP, generates national raster maps of soil properties in corporation with national partners using standardized methodology across countries. Those efforts encourage local laboratories to collect standard soil parameters in a harmonized way, making comparisons and integration of data between countries more and more attainable. An example of advances of the harmonized soil data is the world-wide raster data SoilGrids (Poggio et al., 2021), which were developed by ISRIC based on a huge database of harmonized soil profile (Batjes et al., 2020).

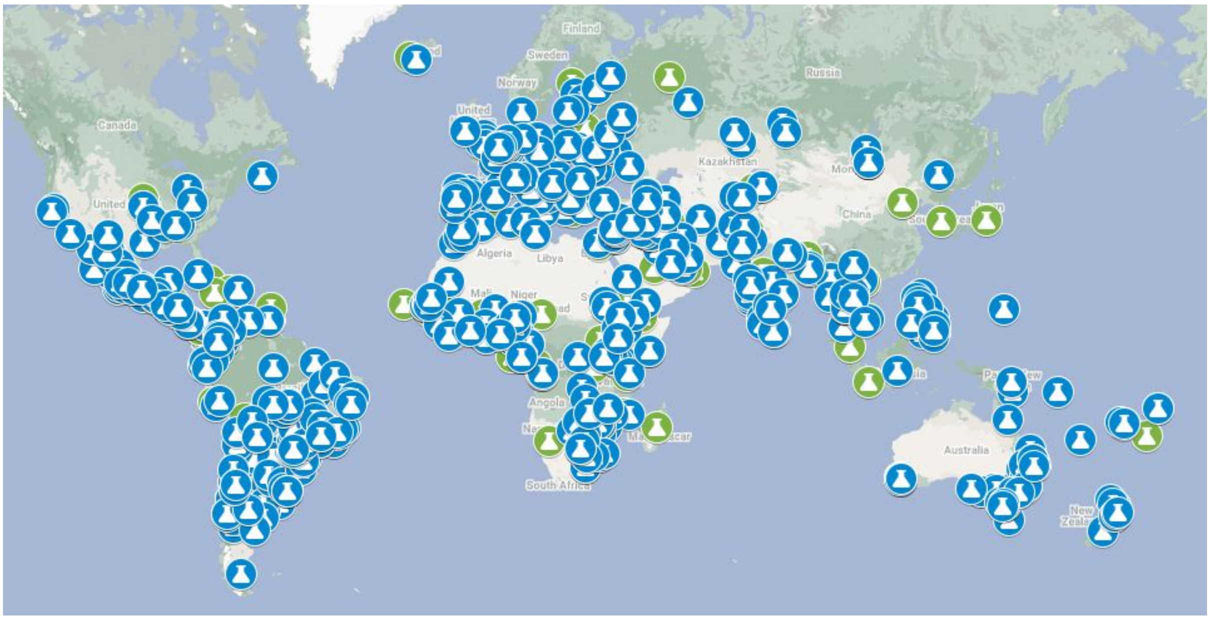

Figure S12. Distribution of soil laboratories registered in GLOSOLAN. Source: https://www.fao.org/global-soil-partnership/glosolan/en

Beside public soil laboratories, there are growing numbers of private soil laboratories which measures soil properties in standardized protocols around the world. For example, soil laboratories of Eurofins (https://www.eurofins.com) are available in Norway, Finland, France, Spain, Germany, Hungary, UK,

Belgium, the Netherlands, Australia and New Zealand, and they measure 20 to 35 soil properties per field, depending on the request of the farmers. Similarly, Agrocares (https://www.agrocares.com) is present in Ukraine, Poland, Hungary, Denmark, Albania, Ivory Coast, Uganda, Kenya, Tanzania, Rwanda, Zambia, Namibia, South Africa, Egypt, North and South Dakota, Minnesota, Mexico, Brazil, Myanmar, Philippines, India and Kazakhstan, measuring more than 60 elements. Most laboratories are wet-chemistry based (which always measure soil organic matter, pH, texture, and major nutrients) or use NIR spectroscopy. With increasing interest in precision farming and accreditation requirements for sustainable agriculture, the reach of the private laboratories is expected to expand and play a more important role in the development of global soil information.

Agronomic knowledge

In principle, any regions with agricultural soil laboratories have some kind of evaluation protocol, because otherwise the measured soil parameter values cannot be assessed and translated into advice for agricultural practices such as fertilization. Many of these protocol are published domestically, usually only in grey literature, making it difficult to scan the existing agronomic knowledge base. Therefore, we limit ourselves here to give a few examples of regional agronomic knowledge, such as:

- guidelines of soil assessment and fertilization for 47 prefectures of Japan assembled by the Japanese ministry of Agriculture, Forestry and fisheries. See this link.
- Guidelines of soil assessment and fertilization in Germany, see here.
- Guidelines of soil assessment and fertilization in France, see here.
- Guidelines of soil assessment and fertilization in tropical soils, see here.
- Guidelines of soil assessment and fertilization in Malawi, see here.
- Guidelines of soil assessment and fertilization in Kenia, see here.
- Guidelines of soil assessment and fertilization in North Dakota, see here.

Beyond the region-specific protocols, efforts have been made to integrate or compare evaluation protocols or fertilization guideline of different regions. For example, the USA consolidated agronomic knowledge basis of 34 states into a database (Lyons et al., 2021). For 18 European countries, the assessment system of P status were reviewed by Jordan-Meille et al. (2012). For West Africa, the international Fertilizer Development Center (IFDC) compiled and updated region- and crop specific fertilization recommendations of many countries and extrapolated that to the whole region (Leenaars et al., 2018).

K. OSI implementation with fewer indicators

The Dutch implementation of OSI requires many indicators, due to ample availability of data and knowledge for Dutch agricultural soil is. However, for regions with relatively poorer availability of data and knowledge, a downsized version of OSI can be implemented.

To explore whether scaling down of OSI yields different results, we applied the OSI on Dutch agricultural sites with fewer numbers of indicators. Here we selected 11 OSI indicators that can be calculated using the basic 10 soil parameters that are mandatory in the GSNmap initiative of FAO: Total N, available P, available K, CEC, pH, clay content, silt content, sand content, organic C, and bulk density. These parameters are usually available on national level, and most of these parameters are also available worldwide from the SoilGrids.

Table S8. Soil indicators used in the full version and downsized version of OSI.

Indicator	Description	Full version	Downsized version
Chemical			
I_C_CEC	Cation Exchange Capacity	1	1
I_C_CU	Copper availability	1	0
I_C_K	Potassium availability	1	1
I_C_MG	Magnesium availability	1	0
I_C_N	Nitrogen availability	1	1
I_C_P	Phosphorus availability	1	1
I_C_PH	Soil acidity	1	1
I_C_S	Sulphur availability	1	0
I_C_ZN	Zinc availability	1	0
Physical			
I_P_CEC	Aggregate stability	1	0
I_P_CO	Compaction	1	0
I_P_CR	Crumbability	1	1
I_P_DS	Drougthstress	1	0
I_P_WS	Wetnessstress	1	0
I_P_DU	Wind erosion	1	1
I_P_SE	Soil sealing	1	1
I_P_WRI	Water retention	1	1
Biological			
I_B_DI	Disease / pest resistance	1	1
I_B_SF	Soil life activity	1	1

The OSI scores calculated with the full version and downsized version of the OSI are shown in Figure 13. Note that environmental and management indicators are not included in the full version, but only chemical, physical, and biological indicators, to be comparable with the downsized version. Therefore, the scores of the full version OSI presented here is slightly different from those presented in the main text Figure 2.

With either version of the OSI, the majority of the Dutch agricultural fields have sufficiently high score (>0.5). Furthermore, the rank order of the OSI scores is reasonably similar between the two versions (rank correlation coefficient 0.60). However, the downsized version of the OSI yields in general higher scores than the full version of OSI: the mean OSI score was 0.77 for the full version and 0.86 for the

downsized version. That is because the poorly performing indicators in the full version, such as soil compaction, S availability, Zn availability, are not included in the downsized version. Thus, the use of more indicators in a soil assessment tool ensures that it covers more aspects of the soil and therefore gives more comprehensive evaluation.

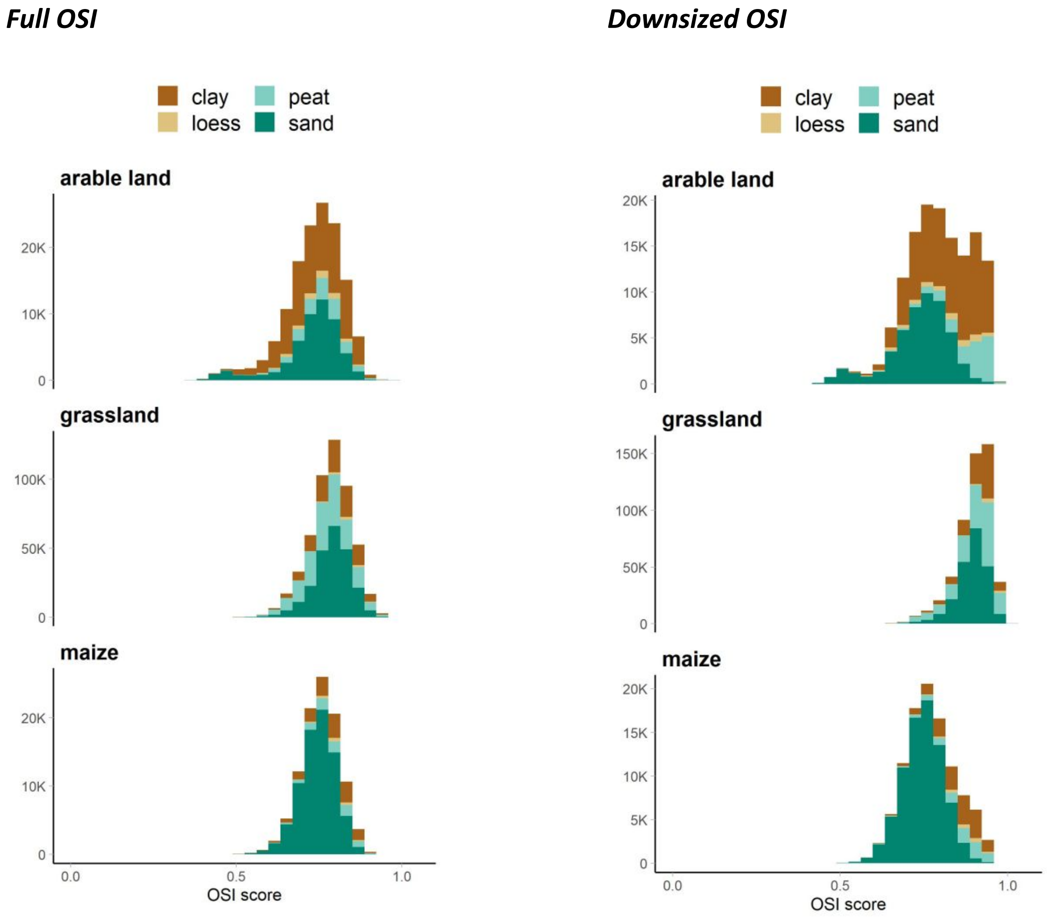

Figure S13. OSI scores of Dutch agricultural fields, evaluated with full version of OSI (left: with 19 soil indicators) and downsized version of OSI (right: with 11 soil indicators).

L. OSI application on 32 fields

Using 32 fields across the Netherlands, we applied the OSI framework to illustrate its relationship with crop yield (Figure S14). Note, that the actual fertilizer, irrigation, and pest management varies across these fields, and that the number of crop-years is also limited, limiting a robust analysis of the actual relationship between soil quality assessments and crop yield. Consequently, this case study has only an illustrative purpose showing that there is likely a positive relationship between soil quality and crop yield (supported for cereals, potato and sugar beets, whereas the number of sites is too low for an indication for maize, onion, and peas). Further validation with field experimental data is ongoing and results will be published later. As discussed in section A each of the underlying algorithms assessing soil functions is derived from field trials linking the individual soil function to the crop response.

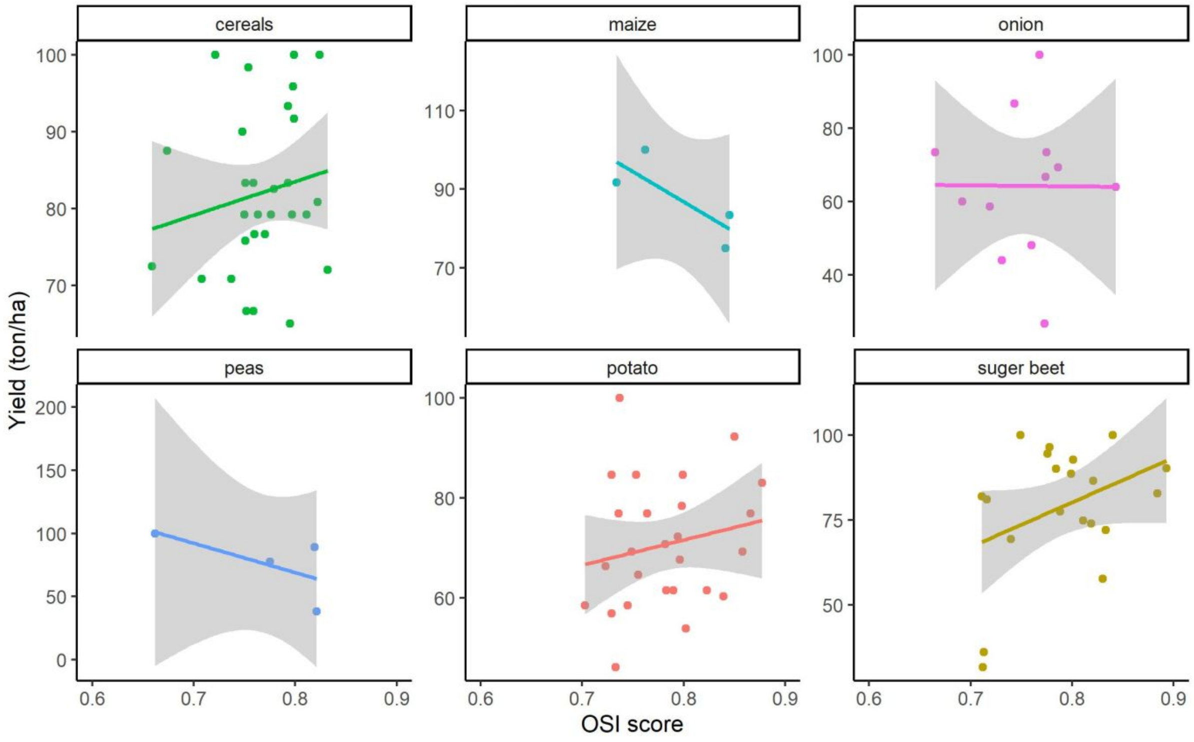

Figure S14. Illustrative examples of relationship between intrative soil quality score and crop yields (where the maximum crop yield is set to 100 for visual purposes) for 6 crop categories.

Supporting References

Andrews, S., Karlen, D., Cambardella, C., 2004. The Soil Management Assessment Framework: A Quantitative Soil Quality Evaluation Method. Soil Sci. Soc. Am. J. 68.

Batjes, N.H., Ribeiro, E., van Oostrum, A., 2020. Standardised soil profile data to support global mapping and modelling (WoSIS snapshot 2019). Earth Syst. Sci. Data 12, 299–320. https://doi.org/10.5194/essd-12-299-2020

Bünemann, E.K., Bongiorno, G., Bai, Z., Creamer, R.E., De Deyn, G., de Goede, R., Fleskens, L., Geissen, V., Kuyper, T.W., Mäder, P., Pulleman, M., Sukkel, W., van Groenigen, J.W., Brussaard, L., 2018. Soil quality – A critical review. Soil Biol. Biochem. 120, 105–125. https://doi.org/10.1016/J.SOILBIO.2018.01.030

Bussink, D., de Haas, M., 2009. Een nieuwe weg in bemestingsonderzoek; resultaten P-PAE 2003-2004 (No. NMI rapport B936_03P). NMI.

Bussink, D.W., van Schöll, L., Van Der Draai, H., van Middelkoop, J.C., Holshof, G., 2014. Naar een herziening van kali-advies grasland Verspreiding Productschap Zuivel (No. NMI-Rapport 1421.N.11). NMI.

Bussink DW, 1998. Mogelijkheden voor een Mg-advies voor klei- en veengrasland (No. NMI-rapport 426.98). NMI.

Bussink, W., van Schöl, L., van der Draai, H., van Riemsdijk, W.H., 2008. Beter waterbeheer en -kwaliteitsmanagement begint op de akker (No. NMI rapport O 1150). NMI.

CBAV, 2021. Handboek bodem en bemesting 2021. Commissie bemesting akkerbouw en vollegrondsgroenten (CBAV) [WWW Document]. URL https://www.handboekbodemenbemesting.nl

CBGV, 2018. Bemestingsadvies. Commissie Bemesting Grasland en Voedergewassen (CBGV), Wageningen.

Debeljak, M., Trajanov, A., Kuzmanovski, V., Schröder, J., Sandén, T., Spiegel, H., Wall, D.P., Van de Broek, M., Rutgers, M., Bampa, F., Creamer, R.E., Henriksen, C.B., 2019. A Field-Scale Decision Support System for Assessment and Management of Soil Functions. Front. Environ. Sci. 7, 1–14. https://doi.org/10.3389/fenvs.2019.00115

Den Boer, D.J., Van Middelkoop, J.C., van Schöll, L., 2010. Interactie tussen N en K op grasland. Herziening kali-advies gewenst? (No. NMI-Rapport 1347.09). NMI.

Den Boer DJ, Reijneveld JA, 1997. Effects of Mg fertilizer type and level on chemical composition of grass on a sandy soil, 1994.

Dontsova, K.M., Norton, L.D., 2002. CLAY DISPERSION, INFILTRATION, AND EROSION AS INFLUENCED BY EXCHANGEABLE Ca AND Mg. Soil Sci. 167.

Ehlert, P., Burgers, S., Steenhuizen, J., Van Lune, P., Loman, H., 1998. De betekenis van grondonderzoek met 0,01 M calciumdikidde als basis voor het kaliumbemestingsadvies van bouwland: Herijking van de kaliumbemestingsadviezen voor akkerbouwland (No. Rapport 87). DLO Instituut voor Agrobiologisch en Bodemvruchtbaarheidsonderzoek.

Ehlert, P., Van Middelkoop, J., Regelink, I., De Haan, J., van Geel, W., 2018. Veeljarige fosfaatproefvelden op gras- en bouwland. Syntheserapport (No. WUR Rapport 2906). Wageningen Environmental Research.

Goselink, G.R.A., van Erp, P.J., 1999. Waardering organischestofgehalten in landbouwgronden (No. NMI-rapport B655.99). NMI.

Groenendijk, P., van Boekel, E., Renaud, L., Greijdanus, A., Michels, R., de Koeijer, T., 2016. Landbouw en de KRW-opgave voor nutriënten in regionale wateren (No. WenR-rapport 2749). Wageningen Environmental Research, Wageningen.

Hanegraaf, M.C., Den Ouden, J.B., Termorshuizen, A.J., Visser, A., Van Schöll, L., Guldemond, A., 2013. Bodemvitaliteit in landbouw- en natuurgebieden in de provincie Noord-Brabant.

Hassink, J., 1995. ORGANIC MATTER DYNAMICS AND N MINERALIZATION IN GRASSLAND SOILS. PhD thesis, Wageningen University.

Heeres, E., Van Erp, P.J., 1999. Relatie tussen organische stof en slempgevoeligheid van landbouwgronden. Meststoffen 44–48.

Huinink, J.T.M., 2018. Bodem/perceel geschiktheidsbeoordeling voor Landbouw, Bosbouw en Recreatie (No. Bodemconsult-Arnhem rapport 2018-3). Bodemconsult-Arnhem, Oosterbeek.

Janssen, B.H., 1984. A simple method for calculating decomposition and accumulation of 'young' soil organic matter. Plant Soil. https://doi.org/10.1007/BF02205588

Jordan-Meille, L., Rubæk, G., Ehlert, P., Genot, V., Hofman, G., Goulding, K., Recknagel, J., Provolo, G., Barraclough, P., 2012. An overview of fertilizer-P recommendations in Europe: Soil testing, calibration and fertilizer recommendations. Soil Use Manag. 28. https://doi.org/10.1111/j.1475-2743.2012.00453.x

Kabata-Pendias, A., Pendias, H., 2001. Trace Elements in Soils and Plants. 3rd Edition. CRC Press, Boca Raton.

Leenaars, J.G.B., Ruiperez González, M., Kempen, B., 2018. Extrapolation of fertilizer nutrient recommendations for major food crops in West Africa; a proof of concept (with dataset), Project report for IFDC, USAID - West Africa Fertilizer Program, Accra. ISRIC, Wageningen, NL.

Lyons, S.E., Arthur, D.K., Slaton, N.A., Pearce, A.W., Spargo, J.T., Osmond, D.L., Kleinman, P.J.A., 2021. Development of a soil test correlation and calibration database for the USA. Agric. Environ. Lett. 6, e20058. https://doi.org/https://doi.org/10.1002/ael2.20058

Marshall, T.J., Holmes, J.W., 1979. Soil Physics. Cambridge University Press, Cambridge.

Moebius-Clune, B.N., Moebius-Clune, D.J., Gugino, B.K., Idowu, O.J., Schindelbeck, R.R., Ristow, A.J., van Es, H.M., Thies, J.E., Shayler, H.A., McBride, M.B., Kurtz, K.S.., Wolfe, D.W., Abawi, G.S., 2016. Comprehensive assessment of soil health – The Cornell Framework, Edition 3. ed. Cornell University, Geneva, NY.

NMI, n.d. Nationaal Agrarisch Bodem Archief [WWW Document]. URL https://www.nmi-agro.nl/works/nationaal-agrarisch-bodem-archief/ (accessed 8.30.22).

Oenema, O., Postma, R., 2003. Managing Sulphur in Agroecosystems. Sulphur in Plants 45–70. https://doi.org/10.1007/978-94-017-0289-8_3

Poggio, L., de Sousa, L.M., Batjes, N.H., Heuvelink, G.B.M., Kempen, B., Ribeiro, E., Rossiter, D., 2021. SoilGrids 2.0: producing soil information for the globe with quantified spatial uncertainty. SOIL 7, 217–240. https://doi.org/10.5194/soil-7-217-2021

Postma, R., Van Dijk, T.A., 2004. Organische stofopbouw en N-mineralisatie; op kernbedrijven; verfijning model MINIP (No. OV0414), Telen met toekomst rapport. Plant Research International.

Rietra, R.P.J.J., Römkens, P.F.A.M., Japenga J., 2004. Cadmium en zink in bodem en landbouwgewassen in de

Kempen (No. Alterra-rapport 974). Alterra.

Rinot, O., Levy, G.J., Steinberger, Y., Svoray, T., Eshel, G., 2019. Soil health assessment: A critical review of current methodologies and a proposed new approach. Sci. Total Environ. 648, 1484–1491. https://doi.org/10.1016/j.scitotenv.2018.08.259

Ros, G.H., 2011. Predicting soil Nitrogen Supply; relevance of soil of extractable soil organic matter fractions. PhD thesis, Wageningen University.

Ros, G H, Bussink, D.W., 2013. Een dynamisch N-advies (No. NMI-rapport 1504.N.13). NMI.

Ros, G.H., Bussink, D.W., 2013. Ontwikkeling rekenregel stuifgevoeligheid (No. NMI-notitie 1504.N.13). NMI, Wageningen.

Ros, G.H., Bussink, W., 2011. Naar een ander K-advies op bouwland, Notitie 1436.N.11. NMI.

Rutgers, M., van Wijnen, H.J., Schouten, A.J., Mulder, C., Kuiten, A.M.P., Brussaard, L., Breure, A.M., 2012. A method to assess ecosystem services developed from soil attributes with stakeholders and data of four arable farms. Sci. Total Environ. 415, 39–48. https://doi.org/10.1016/J.SCITOTENV.2011.04.041

Sluijsmans, C., 1987. Het magnesiumgehalte van weidegras in afhankelijkheid van bemesting en bodemvruchtbaarheid (No. IB-rapport 1-87). Instituut voor Bodemvruchtbaarheid, Haren.

Sluijsmans, C., 1967. INVLOED VAN BEMESTING MET KIESERIET EN KALIZOUT OP HET MAGNESIUMGEHALTE VAN WEIDEGRAS (No. Gestencilde verslagen van interprovinciale proeven Nr. 120). PAW, Lelystad.

Thoumazeau, A., Bessou, C., Renevier, M.-S., Panklang, P., Puttaso, P., Peerawat, M., Heepngoen, P., Polwong, P., Koonklang, N., Sdoodee, S., Chantuma, P., Lawongsa, P., Nimkingrat, P., Thaler, P., Gay, F., Brauman, A., 2019a. Biofunctool®. Ecol. Indic. 97, 429–437. https://doi.org/10.1016/j.ecolind.2018.10.028 T4 - A new framework to assess the impact of land management on soil quality. Part B: investigating the impact of land management of rubber plantations on soil quality with the Biofunctool® index M4 - Citavi

Thoumazeau, A., Bessou, C., Renevier, M.-S., Trap, J., Marichal, R., Mareschal, L., Decaëns, T., Bottinelli, N., Jaillard, B., Chevallier, T., Suvannang, N., Sajjaphan, K., Thaler, P., Gay, F., Brauman, A., 2019b. Biofunctool®. Ecol. Indic. 97, 100–110. https://doi.org/10.1016/j.ecolind.2018.09.023 T4 - A new framework to assess the impact of land management on soil quality. Part A: concept and validation of the set of indicators M4 - Citavi

Van Bakel, J., Huinink, J., Van Der Bolt, F.J.E., 2005. HELP-2005, uitbreiding en actualisering van de HELP-tabellen ten behoeve van het Waternood-instrumentarium (No. Rapport / STOWA; No. 2005 16). Stowa.

van den Akker, J.J.H., de Vries, F., Vermeulen, G.D., Hack-ten Broeke, M.J.D., Schouten, T., 2012. Risico op ondergrondverdichting in het landelijk gebied in kaart (No. Alterra-rapport 2409). Alterra, Wageningen.

Van Der Wal, A., de Lijster, E., Dijkman, W., 2016. Ontwerp Label Duurzaam Bodembeheer (No. CLM-rapport 910). CLM.

Van Kerckhoven, S., Riksen, M., Cornelis, W., 2007. Afbakening van gebieden gevoelig aan winderosie in Vlaanderen (No. BOD/STUD/2007/02, Eindrapport, Universiteit Gent). Vakgroep Bodembeheer, Gent.

van Rotterdam, A.M.D., 2010. The potential of soils to supply phosphorus and potassium processes and predictions. PhD thesis, Wageningen University.

van Rotterdam, A.M.D., Bussink, D.W., Temminghoff, E.J.M., van Riemsdijk, W.H., 2012. Predicting the potential of soils to supply phosphorus by integrating soil chemical processes and standard soil tests. Geoderma 189–190, 617–626. https://doi.org/10.1016/J.GEODERMA.2012.07.003

van Rotterdam, D., Bussink, D.W., Reijneveld, J.A., 2014. Improved Phosphorus Fertilisation Based on Better Prediction of Availability in Soil, in: IFS PROCEEDINGS 755. p. 24.

van Rotterdam, D., Bussink, W., 2016. Fosfaatstreeftoestand in de bodem voor maïs en gras. notitie Bemestingsadvies. CBGV.

van Wijnen, H.J., Rutgers, M., Schouten, A.J., Mulder, C., de Zwart, D., Breure, A.M., 2012. How to calculate the spatial distribution of ecosystem services — Natural attenuation as example from The Netherlands. Sci. Total Environ. 415, 49–55. https://doi.org/https://doi.org/10.1016/j.scitotenv.2011.05.058

Verweij, S., Ros, G., Fujita, Y., Riechelman, W., 2022. OBIC: Calculate the Open Bodem Index (OBI) Score. R package version 2.0.2. [WWW Document]. URL https://github.com/AgroCares/Open-Bodem-Index-Calculator

Vroon HRJ, 2007. Bodemgeschiktheid voor akker- en weidebouw en het vervaardigen van ruilklassenkaarten voor de waardebepaling van de gronden in het landinrichtingsgebied Wintelre-Oerle.

Wienhold, B.J., Karlen, D.L., Andrews, S.S., Stott, D.E., 2009. Protocol for indicator scoring in the soil management assessment framework (SMAF). Renew. Agric. Food Syst. 24, 260–266.

Wösten, J.H.M., De Vries, F., Hoogland, T., Massop, H.T.L., Veldhuizen, A.A., Vroon, H.R.J., Wesseling, J.G., Heijkers, J., Bolman, A., 2013. BOFEK2012, de nieuwe bodemfysische schematisatie van Nederland (No. Alterra-rapport 2387). Alterra, Wageningen.

Wösten, J.H.M., Lilly, A., Nemes, A., Le Bas, C., 1999. Development and use of a database of hydraulic properties of European soils. Geoderma 90, 169–185. https://doi.org/10.1016/S0016-7061(98)00132-3

Wösten, J.H.M., Veerman, G.., de Groot, W.J.., Stolte, J., 2001. Waterretentie- en doorlatendheidskarakteristieken van boven- en ondergronden in Nederland: de Staringreeks (No. Alterra-rapport 153), Alterra Rapport. Alterra, Wageningen. https://doi.org/153
